# An Efficient and Flexible Bifunctional Dual-Band Electrochromic Device Integrating with Energy Storage

**DOI:** 10.1007/s40820-024-01604-0

**Published:** 2024-12-27

**Authors:** Zekun Huang, Yutao Peng, Jing Zhao, Shengliang Zhang, Penglu Qi, Xianlin Qu, Fuqiang Yan, Bing Ding, Yimin Xuan, Xiaogang Zhang

**Affiliations:** 1 Jiangsu Key Laboratory of Electrochemical Energy Storage Technologies, College of Materials Science and Technology, Nanjing University of Aeronautics and Astronautics, Nanjing, 210016 People’s Republic of China; 2 Key Laboratory of Thermal Management and Energy Utilization of Aviation Vehicles, College of Energy and Power Engineering, Nanjing University of Aeronautics and Astronautics, Nanjing, 210016 People’s Republic of China; 3 Center for Microscopy and Analysis, Nanjing University of Aeronautics and Astronautics, Nanjing, 211106 People’s Republic of China

**Keywords:** Electrochromic, Dual-band electrochromic devices, Spectral-selective modulation, Flexible, Energy storage

## Abstract

Dual-band electrochromic devices capable of the spectral-selective modulation of visible (VIS) light and near-infrared (NIR) can notably reduce the energy consumption of buildings and improve the occupants' visual and thermal comfort. However, the low optical modulation and poor durability of these devices severely limit its practical applications. Herein, we demonstrate an efficient and flexible bifunctional dual-band electrochromic device which not only shows excellent spectral-selective electrochromic performance with a high optical modulation and a long cycle life, but also displays a high capacitance and a high energy recycling efficiency of 51.4%, integrating energy-saving with energy-storage. The nanowires structure and abundant oxygen-vacancies of oxygen-deficient tungsten oxide nanowires endows it high flexibility and a high optical modulation of 73.1% and 85.3% at 633 and 1200 nm respectively. The prototype device assembled can modulate the VIS light and NIR independently and effectively through three distinct modes with a long cycle life (3.3% capacity loss after 10,000 cycles) and a high energy-saving performance (8.8 °C lower than the common glass). Furthermore, simulations also demonstrate that our device outperforms the commercial low-emissivity glass in terms of energy-saving in most climatic zones around the world. Such windows represent an intriguing potential technology to improve the building energy efficiency.

**Supplementary Information:**

The online version contains supplementary material available at 10.1007/s40820-024-01604-0.

## Highlights

- A flexible dual-band electrochromic device with a high optical modulation and a long cycle life was reported.
- The device assembled can modulate the visible light and near-infrared independently and effectively, showing higher energy-saving performance than commercial low-emissivity glass in most climatic zones around the world.
- The flexible device also shows good energy storage and energy recycling performances, recycling 51.4% of the energy consumed in the coloration process for local reusing.

**Supplementary Information:**

The online version contains supplementary material available at 10.1007/s40820-024-01604-0.

## Graphical abstract

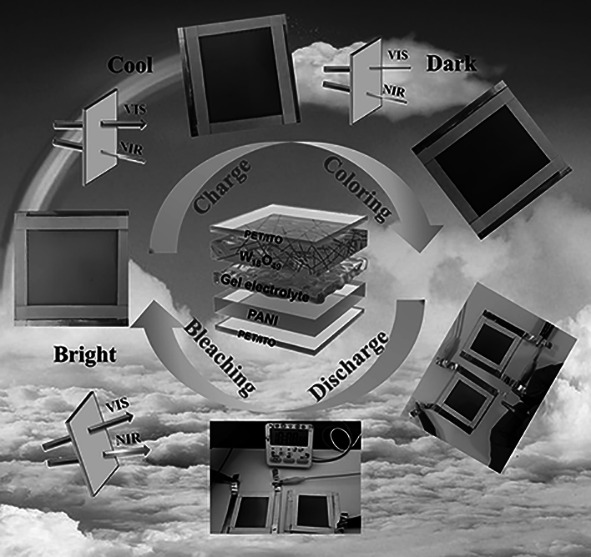

## Introduction

Buildings consume ~ 40% of global energy consumption, and as high as ~ 50% of this energy is presently used for indoor thermal and visual management (e.g., heating, cooling and lighting) [1, 2]. Windows, as the primary means of energy exchange between the interior and exterior environments, play an important role in building energy efficiency because that 20%–40% of energy used in heating and cooling is lost through windows [3–5]. It is therefore imperative to develop energy-efficient windows for green and zero-carbon buildings. Electrochromic smart windows have emerged as an attractive technology to reduce the building’s energy consumption by up to 20% than common windows through dynamically and reversibly modulating the transmittance of solar radiation [6–19].

The solar radiation consists of ultraviolet (UV), visible light (VIS, 400–780 nm) and near-infrared (NIR, 780–2500 nm), and the NIR energy accounts for ~ 43% of the total solar irradiance [20–24]. Therefore, the NIR modulation and NIR-selective modulation of the smart windows have a remarkable effect on the energy consumption, and indoor occupant comfort of the buildings [25–27]. However, many of the reported electrochromic smart windows can only provide VIS modulation, or block VIS and NIR without any spectral selectivity. Dual-band electrochromic smart windows, which could modulate the VIS and NIR independently and dynamically through bright, cool and dark modes, have been introduced as a promising energy-efficient technology for improving the building energy efficiency [28–39]. The energy consumption of buildings can be further lower up to 10%–20% than traditional electrochromic smart windows through the smart regulation of solar light and solar heat into buildings.

During the past decade, substantial progress has been made in dual-band electrochromic smart window technology [25–27]. The dual-band electrochromic performance such as spectral-selectivity and switching speed have been improved by using advanced composites and single-component nanomaterials [28–39]. Recently, Li et al. [34] reported a novel dual-band zinc anode-based electrochromic device integrating electrochromic window with Internet of Things (IoT), which could continuously transport solar energy to indoor appliances by remotely controlling the repeated bleached-tinted cycles during the daytime, providing an intriguing potential technology for green buildings. Cao et al. [2] recently reported a tri-band electrochromic smart window that combines spectral-selective modulation of VIS and NIR with radiative cooling of mid-infrared, that significantly enhance the energy-saving performance and provide a new window design for carbon neutrality. Despite these advancements, the development of the current dual-band electrochromic smart windows is still impeded by the issues of low optical modulation and poor durability of the device.

In this study, we demonstrate an efficient and flexible bifunctional dual-band electrochromic device (DBED) based on oxygen deficient tungsten oxide nanowires (W_18_O_49_ NWs), which not only displays an excellent spectral-selective electrochromic performance with a high optical modulation and a long cycle life, but also shows a good energy storage and efficient energy recycling performance. The single-component oxygen-deficient WO_3−x_ [33] have shown superior dual-band electrochromic performance than other previously reported dual-band electrochromic materials [28–39], showing great potential in the pratical applications. The W_18_O_49_ NW was therefore selected as electrochromic cathode material due to its nanowire structure, abundant oxygen-vacancies and high surface area, which endow it high flexibility, tunable localized surface plasmon resonance (LSPR) in the NIR range and adequate ion storage sites. Consequently, the W_18_O_49_ NW film delivers impressive dual-band electrochromic performance including high optical modulation (73.1% at 633 nm and 85.3% at 1,200 nm), high spectral-selectivity and fast switching speed. The porous polyaniline (PANI) was selected as anode due to its high conductivity, high flexibility and good electrochromic performance. The porous PANI anodes not only can provide an ion storage function to balance the cathode charge and reduce the operating voltage of the device, but also are able to enhance the optical modulation performance of the device. The prototype flexible DBED assembled by W_18_O_49_ NW cathode and polyaniline anode is able to modulate the VIS light and NIR independently and effectively through three distinct modes with high optical modulation, fast response speed, and long cycle life (3.3% capacity loss after 10,000 cycles) which can be attributed to the high structure stability of monoclinic W_18_O_49_ during Li^+^ intercalation/de-intercalation without any phase transition. We experimentally demonstrate the high energy-saving performance of the device with 8.8 °C lower than the common glass under simulated sunshine. Furthermore, simulations also demonstrate that the device outperforms the commercial low-emissivity glass in terms of energy-saving in most climatic zones around the world. The flexible device also shows good energy storage performance with a high capacitance of 36.7 F m^−2^ and can recycle 51.4% of the energy consumed in the coloration process for local reusing. Thus, the net energy consumption of the device in a round-trip electrochromic operation is reduced to only 24.5 mWh m^−2^. The flexible bifunctional DBED with high spectral-selectivity electrochromic performance, good energy storage property and efficient energy recycling demonstrated here is an energy-efficient technology to reduce the buildings’ energy consumption, contributing significantly to the global carbon neutrality and sustainability goals.

## Experimental Section

### Materials

Propylene carbonate (PC, anhydrous, 99.7%), lithium perchlorate (LiClO_4_, ≥ 95%), tributylmethylammonium (TMA) bis(trifluoromethanesulfonyl)imide (98%), aniline and polymethacrylate (PMMA) were purchased from Aladdin. Acetone, isopropyl alcohol, anhydrous ethanol and tungsten hexachloride (WCl_6_, ≥ 99%) were purchased from Macklin. Concentrated sulfuric acid (H_2_SO_4_, 70%) was obtained from Nanjing Reagent. All chemicals were used as received. Polyethyleneterephthalate/ Sn-doped indium oxide (PET/ITO) flexible substrate (20 × 30 cm^2^, 0.175 mm, < 35 Ω sq^−1^) and normal glasses were supplied by Kaivo.

### Preparation of W_18_O_49_ NWs

W_18_O_49_ NWs were prepared by solvothermal method and 0.06 g WCl_6_ was dissolved in 40 mL absolute anhydrous ethanol. The solution is then transferred to a teflon-lined autoclave where it is heated at 180 °C for 24 h and the autoclave is finally cooled to room temperature and centrifuged. The obtained blue product was centrifuged with anhydrous ethanol at 10,000 rpm for 5 min and cleaned twice, and dispersed in 10 mL anhydrous ethanol with a concentration of 3 mg mL^−1^.

### Preparation of W_18_O_49_ NWs Films

The diluted W_18_O_49_ NWs solution with a concentration of 1 mg mL^−1^ was used for ultrasonic spraying at a flow rate of 0.1 mL min^−1^, a step rate of 50 mm min^−1^, a spraying height of 25 mm and a spraying width of 1.5 mm to prepare W_18_O_49_ nanowires with good uniformity.

### Preparation of PANI Films

The PANI film was prepared by electrodeposition with three electrodes. The counter electrode is 2 × 2 cm^2^ platinum foil, reference electrode Ag/AgCl electrode, working electrode flexible PET/ITO substrate. The electrolyte is configured by adding 5.3 mL of concentrated sulfuric acid and 4.6 mL of aniline to 90.1 mL of water and mixing evenly until it is pale brown and transparent. A constant potential of 0.75 V (vs. Ag/AgCl) was applied using a Koster 310X electrochemical workstation. The PANI film was prepared by continuous electrodeposition for 5 min at an applied potential of 0.75 V. After the electrodeposition was completed, the film was rinsed with deionized water, dried in the air, and placed in a vacuum drying oven at 60 °C for use.

### Preparation of Electrolyte

1 M LiClO_4_/PC was used as electrolyte to investigate the electrochromic and electrochemical properties of the film. Add 5.32 g of LiClO_4_ to 50 mL of PC solution and stir until LiClO_4_ is completely dissolved. The gel electrolyte was prepared by adding 10% PMMA (mass ratio) into 1 M LiClO_4_/PC electrolyte, then heated and stirred at 80 °C until PMMA was completely dissolved.

### Assembly of DBED

The DBED was assembled by using a W_18_O_49_ NWs film (7 × 7 cm^2^) as the cathode, a PANI film (7 × 7 cm^2^) as the anode, and a 1 M LiClO_4_/PC gel as the electrolyte. The two facing electrodes of the optical cell were spaced apart by a ~ 1 mm thick 3 M double-sided tape. The electrolyte was introduced to the cell cavity with a syringe and the cell was sealed by a UV glue.

### Materials Characterizations

X-ray diffraction (XRD) patterns were measured by PANalytical Empyrean diffractometer. The morphology of W_18_O_49_ NWs was characterized by transmission electron microscopy (TEM) on a Thermo Fisher spectra 300 microscope. X-ray photoelectron spectroscopy (XPS) analysis was performed on KRATOS AXIS SUPRA. The Ramon spectra of W_18_O_49_ NWs were measured by LabRAM HR Evolutio. Nitrogen adsorption/desorption isotherms of W_18_O_49_ NWs was test by BET, ASAP 2020. The presence of oxygen vacancies in W_18_O_49_ NWs was determined by a Bruker BioSpin GmbH E500 electron EPR. The nanostructure of W_18_O_49_ NW film was analyzed by field-emission SEM (FESEM, Hitachi S-4800). The VIS–NIR transmittance spectra were recorded by a PerkinElmer LAMBDA 1050 + UV–VIS-NIR spectrophotometer.

### Electrochemical and Electrochromic Measurements

The electrochemical and electrochromic properties of W_18_O_49_ NW films were investigated in situ by a spectroelectrochemical cell in the three-electrode configuration. The W_18_O_49_ nanowire film was used as a working electrode and the counter electrode and the reference electrode were Pt foil and an Ag/AgCl electrode respectively. The electrolyte was 1 M LiClO_4_/PC. Cyclic voltammetry (CV) measurement was performed on a Corrtest310X electrochemical workstation. In situ optical transmittance spectra as a function of the applied potentials were recorded by a Lambda 1050 + UV/VIS/NIR spectrometer. Meanwhile, the transmittance of the PET-ITO in the same electrolyte was used as the baseline. All potentials in three-electrode measurements were quoted with respect to an Ag/AgCl reference.

Switching time is defined as the time required to reach 90% of the full optical modulation in the specified potential. The diffusion coefficient *D* (cm^2 ^s^−1^) of Li^+^ was calculated by Randles Sevcik equation: *I*_*p*_ = 2.69 × 10^5^ ACD^1/2^n^3/2^ υ^1/2^, where *I*_*p*_ (A) is the peak current, A (cm^2^) is the area of the electrode, C (mol cm^−3^) is the concentration of Li^+^ in the bulk solution (electrolyte), and n is the number of electrons involved in the redox process, υ (V s^−1^) is the scan rate. Coloration efficiency (CE) was calculated from the formula: CE = Δ*OD*/Δ*Q* = log(*T*_b_/*T*_c_)/Δ*Q*, where Δ*Q* is the injected charge, *T*_b_ and *T*_c_ are the transmittances in the bleached and colored states at the specified wavelength respectively. The areal capacitance is calculated according to the following equation: C = *I*Δt/*S*Δ*V*, where *C* (F cm^−2^) is the areal capacitance,* I* (mA) represents the discharge current, and* S* (cm^2^), Δ*V* (V) and Δ*t* (s) designate the area of active materials, potential windows excluding the IR voltage drop and total discharge time, respectively.

For the 2-electrode device measurements, in situ optical transmission spectra and real-time transmittance measurements were recorded as a function of the applied voltages on Lambda 1050 + UV/VIS/NIR spectrometer. The background transmittance of the air atmosphere was used as the baseline. Cyclic stability was tested at 0.1 mA cm^−2^ for 10,000 cycles. Energy input and output (E, Wh m^−2^) was calculated based on the equation: E = $\int \text{U I dt}$, where U (V) is the voltage, *I* is the current density (A m^−2^), *t* (h) is the time.

### EnergyPlus Simulation of Energy-Saving

EnergyPlus is utilized for comprehensive energy simulation, employing an Ideal Loads Air System. The building in question measures 20 m (L) × 10 m (W) × 3 m (H). The glazing system covers a total area of 48 m^2^, accounting for a 32% window-to-wall ratio. The internal gains and HVAC systems have been meticulously designed within the simulation. The indoor temperature is set according to the actual situation, with hourly weather data from a typical meteorological year used for external conditions. From 20:00 to 07:00, the indoor lighting is consistently set to 0 W m^−2^. During the daytime, the illuminance is set to 500 lx. When the daytime illuminance exceeds 500 lx, the lighting power remains at 0; otherwise, it increases linearly from 0 to 8 W m^−2^ as the illuminance decreases below this threshold. The HVAC energy consumption was calculated for both the baseline building model with conventional wall properties (as set in the downloaded EnergyPlus models) and the model with modified wall surface optical properties, informed by our experimentally determined material values and obtained using the EnergyPlus software. By comparing the energy usage between these models, we determined the annual energy savings for cooling, heating, interior lighting, and total HVAC systems. The energy consumption for heating and cooling is calculated based on formulas: *E*_H_ = *Q*_H_/(*A*η_1_q_1_q_2_) and *E*_C_ = *Q*_C_/(*A* × COP_C_), where *Q*_H_ (MJ) is accumulated thermal energy consumption, *A* (m^2^) is the area of windows, η_1_ is the comprehensive efficiency of a heating system with a coal-fired boiler as the heat source taken as 0.81, q_1_ (MJ kgce^−1^) is the calorific value of standard coal taken as 8.14 MJ kgce^−1^, q_2_ (kgce MJ^−1^) is the comprehensive coal consumption for power generation taken as 0.330 kgce MJ^−1^, COP_C_ is the comprehensive performance coefficient of the cooling system in public buildings taken as 3.50. To evaluate the energy-saving performance across various climates, we analyzed data from 18 cities globally, representing different climate zones.

## Results and Discussion

### Preparation and Characterization of the W_18_O_49_ NWs

**Fig. 1:**
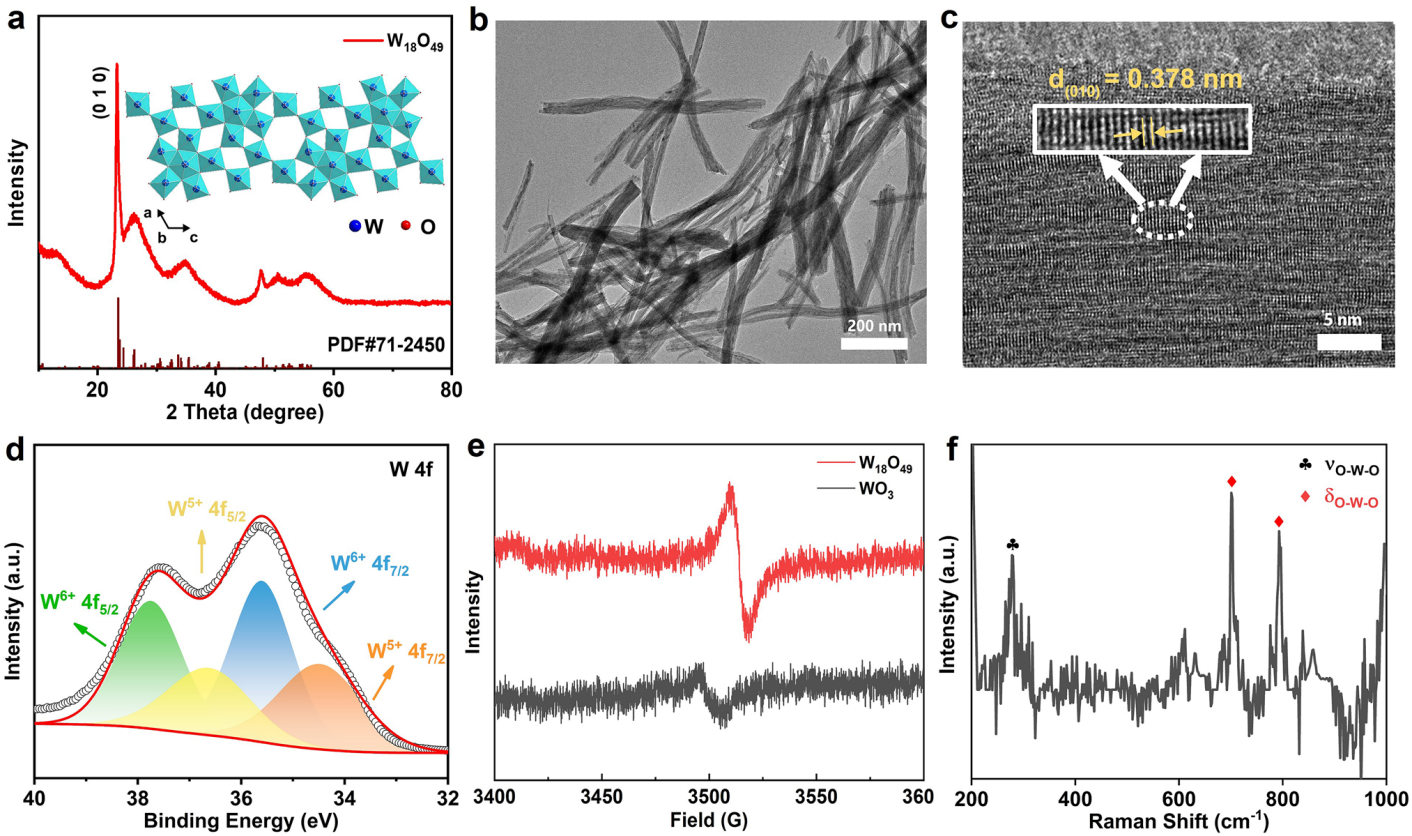
**a** XRD pattern of W_18_O_49_ NWs. **b** TEM and **c** HRTEM images of W_18_O_49_ NWs. **d** W 4*f* XPS spectra, **e** EPR signal and **f** Raman spectrum of W_18_O_49_ NWs

The W_18_O_49_ NWs were synthesized by an optimized solvothermal method (details in the Experimental Section) [6, 40]. X-ray diffraction (XRD, Fig. 1a) confirms the synthesized W_18_O_49_ NWs as phase-pure monoclinic W_18_O_49_ (JCPDS No. 71-2450). Such monoclinic structure of W_18_O_49_ exhibits a large open tunnel formed by WO octahedra that facilitates ion diffusion. The transmission electron microscopy (TEM, Fig. 1b) image reveals that the prepared W_18_O_49_ has a nanowire morphology with a diameter of ~ 40 nm. The high-resolution transmission electron microscopy (HRTEM, Fig. 1c) image shows the lattice spacing of 0.378 nm corresponding well with the interplanar distance of the W_18_O_49_ (010) planes, which not only confirms the XRD findings but also suggests [010] as the NW growth direction. The nanowire structure of W_18_O_49_ also endows it a relatively high surface area of 64.7 m^2^ g^−1^ (Fig. S1), which is advantageous for Li^+^ absorption. The X-ray photoelectron spectroscopy (XPS) survey spectrum (Fig. S2a) of the sample confirms only the presence of W and O elements and the W 4*f* XPS spectrum (Fig. 1d) can be deconvoluted into two pairs of peaks demonstrating the co-existence of W^6+^ and W^5+^. The peaks at 35.60 and 37.75 eV correspond to the W 4*f*_7/2_ and W 4*f*_5/2_ peaks of W^6+^, and the peaks at 34.50 and 36.65 eV correspond to the W 4*f*_7/2_ and W 4*f*_5/2_ peaks of W^5+^ respectively. As shown in Fig. S2b, the deconvolution analysis of the O 1*s* peak indicates that it can be divided into three distinct peaks, with the peak at 530.66 eV corresponding to lattice oxygen, the peak at 531.74 eV attributed to oxygen vacancies, and the peak at 533.25 eV related to adsorbed oxygen. The large area ratio of the oxygen vacancy peak further confirms the presence of oxygen vacancies in W_18_O_49_ NWs. The electron paramagnetic resonance (EPR) spectroscopy, an advanced but straightforward tool for the detection of unpaired electrons, was also conducted to confirm the presence of oxygen vacancies. The W_18_O_49_ NWs showed a symmetric EPR signal at a G factor of 2.0023 (Fig. 1e) which is assigned to the trapping of unpaired electrons at oxygen vacancies. The stronger EPR signal intensity of the W_18_O_49_ NWs than that of WO_3_ indicates an abundance of oxygen vacancies. The abundant oxygen vacancies of W_18_O_49_ NWs provide large space for ion intercalation and diffusion, which not only enhances the ion diffusion coefficient, but also mitigates the lattice distortion induced by ion intercalation and thus improving the material's cyclic stability. Furthermore, these vacancies increase the concentration of free electrons, which strengthens the LSPR effect in the NIR region, enabling the dual-band electrochromic functionality.The Raman spectrum (Fig. 1f) reveals three principal peaks at frequencies of 278, 701, and 793 cm⁻^1^. The peak at 278 cm⁻^1^ is attributed to the stretching vibration mode of the O-W–O framework, while the peaks at 701 and 793 cm⁻^1^ correspond to the bending vibration modes of the O–W–O framework.

### Electrochemical and Electrochromic Properties of the W_18_O_49_ NW Films

**Fig. 2:**
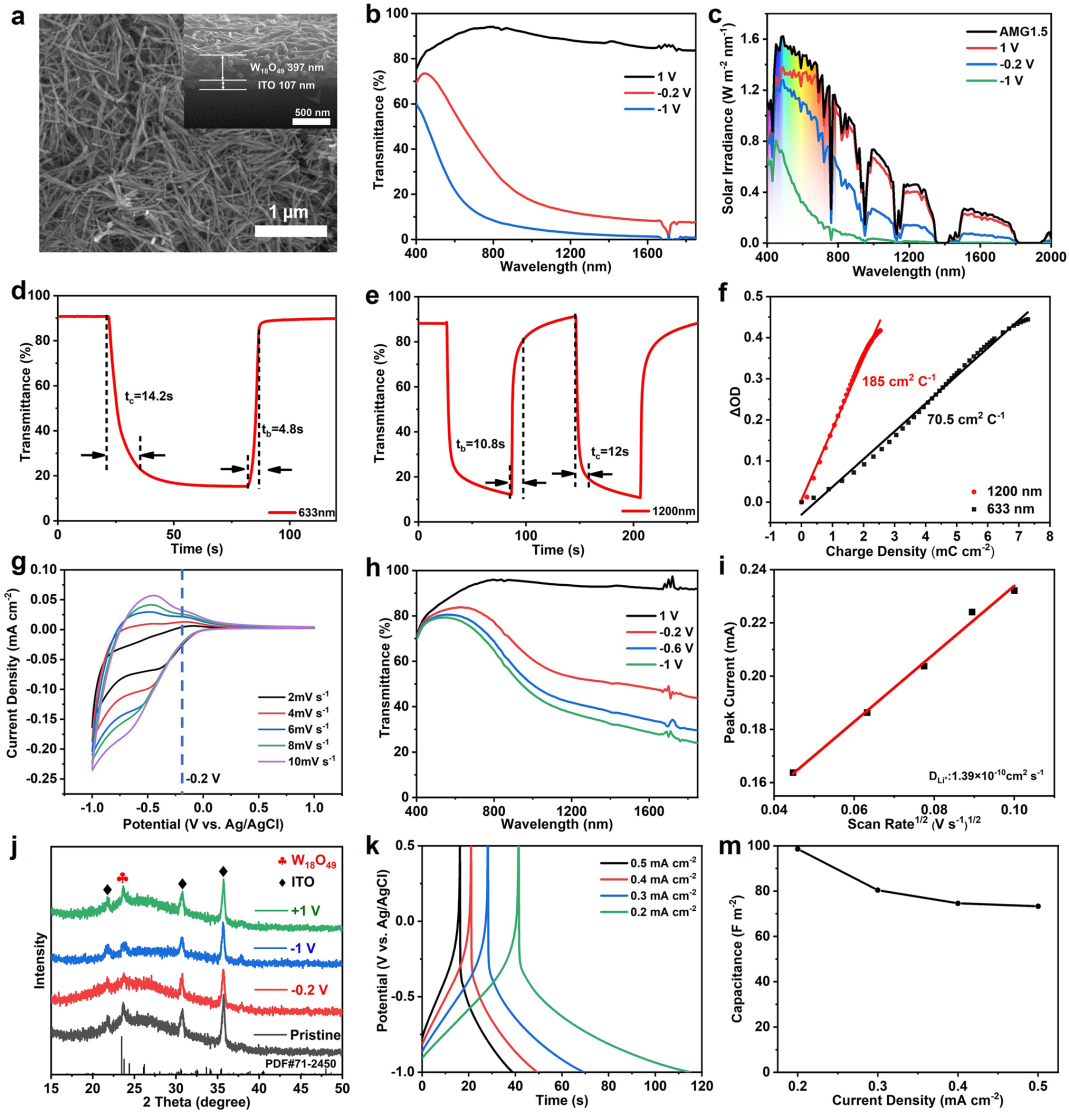
**a** Surface and cross-sectional (inset) SEM images of W_18_O_49_ NW films. **b** Optical transmittance spectra and **c** solar irradiance spectra of W_18_O_49_ NW film in the bright, cool and dark modes, respectively. **d** Real-time transmittance spectra at 633 nm and **e** at 1200 nm.** f** Optical density changes of W_18_O_49_ NW films as a function of injected charge density.** g** Cyclic voltammograms of W_18_O_49_ NW film at different scan rates. **h** Transmittance spectra of W_18_O_49_ NW film in 0.1 M TMA^+^/PC electrolyte at different applied potentials. **i** The corresponding cathodic peak current as a function of the square root of scanning rates. **j** Ex situ XRD patterns of a W_18_O_49_ NW film in the pristine state and at − 0.2, − 1, and + 1 V. **k** The galvanostatic charge/discharge curves of W_18_O_49_ NW film at different current densities. **m** Areal capacitance as a function of current density

The flexible W_18_O_49_ NW films were prepared by ultrasonic spraying method (details in the Experimental Section). The W_18_O_49_ NW films with spaying 100 times displayed the best optical modulation, with high optical modulation of 73.1% at 633 nm and 85.3% at 1,200 nm, outperforming those of W_18_O_49_ NW film with 50 spraying times (52.7% at 633 nm and 79% at 1,200 nm) and 150 spraying times (59% at 633 nm and 66.5% at 1,200 nm) as shown in the Fig. S3. Thus, the W_18_O_49_ NW films with spaying 100 times were adopted in our following studies. Scanning electron microscopy (SEM, Fig. 2a) showed that the nanowire morphology of W_18_O_49_ was maintained after the film formation process, and the cross-sectional SEM image depicted that the thickness of the W_18_O_49_ NW film was ~ 397 nm (Fig. 2a inset). The laser confocal microscopy (Fig. S4) confirmed that the of W_18_O_49_ NW thin films had a good surface uniformity.

The electrochemical and electrochromic properties of W_18_O_49_ NW thin films were investigated in situ by a spectroelectrochemical cell operating in the three-electrode configuration (with a Pt foil counter electrode, an Ag/AgCl reference electrode, and a 1 M LiClO_4_/propylene carbonate electrolyte). As shown in Fig. 2b, the optical transmittance spectra of the W_18_O_49_ NW films display an impressive dual-band electrochromic performance. The W_18_O_49_ NW films also show a high optical modulation of 73.1% and 85.3% at 633 and 1200 nm respectively, which are higher than most of the dual-band electrochromic materials in the current literature (Table S1) [28–39]. Moreover, the VIS and NIR transmittance can also be independently and effectively regulated with three distinct modes by controlling the applied potentials. At 1 V, the W_18_O_49_ NW film is in the “bright” mode which is fully transparent in the VIS and NIR. At the reduced applied potential of -0.2 V, the “cool” mode is activated where the W_18_O_49_ NW thin film blocks 85.7% of the NIR while maintaining a good VIS transmittance of 62.5% (Table 1). Since solar energy is not uniformly distributed across all wavelengths (especially in the NIR region), the actual solar irradiance spectra of the W_18_O_49_ NW films (Fig. 2c) in the three operating modes were calculated to better reflect the application performance in solar irradiation modulation. In the cool mode (− 0.2 V), the W_18_O_49_ NW film is able to block 63.5% of solar heat in the NIR range (780–1850 nm) for building heat load reduction while providing a high VIS light transmittance of 72.5% for daylighting (Table 1). The building energy consumption on air-conditioning and lighting can therefore be lowered significantly by operating the W_18_O_49_ NW film in the cool mode. In the dark mode (− 1 V), the W_18_O_49_ NW film blocks most of the VIS light (72.2%) and solar heat (95.9%) into the building, which is suitable for personal privacy protection and the further reduction of the building solar heat gain.

**Table 1:** Integrated optical transmittance (T) and integrated solar irradiance transmittance (T′) of W_18_O_49_ NW film in the VIS light (400–780 nm), NIR (780–1850 nm); and total solar irradiation (sol, 400–1850 nm) at 1, − 0.2 and − 1 V

Mode	T_VIS_ (%)	T_NIR_ (%)	Tsol (%)	T_VIS_′ (%)	T_NIR_′ (%)	Tsol′ (%)
Bright (1 V)	88.8	89.5	87.3	88.9	89.8	89.2
Cool (− 0.2 V)	62.5	16.3	22.3	72.5	36.5	57.6
Dark (− 1 V)	27	4.2	8.2	27.8	4.1	18.1

The calculations are based on the following equations: $\text{Tsol}=\frac{\int T\left(\lambda \right)d\lambda }{\int d\lambda }$, ${\text{Tsol}}^{\prime }  = \frac{{\int {\text{T}} \left( \lambda  \right)\psi (\lambda ){\text{d}}\lambda }}{{\int \psi  (\lambda ){\text{d}}\lambda }}$, where $T(\lambda )$ is the transmittance at wavelength of $\lambda$, and $\psi (\lambda )$ is the solar irradiance at 1.5 air mass

The real-time optical transmittance measurements were conducted to characterize the switching speeds of the W_18_O_49_ NW film between different operating modes (bright, cool and dark). Figure 2d shows the coloration (t_c_) and bleaching (t_b_) times between the bright (1 V) and dark (− 1 V) modes measured at 633 nm are 14.2 and 4.8 s, while the t_c_ and t_b_ between bright (1 V) and cool (− 0.2 V) modes measured at 1200 nm are 12.0 and 10.8 s (Fig. 2e) respectively. The fast switching speed of the W_18_O_49_ NW film can be ascribed to Li^+^ fast transport brought by the nanowire structure and abundant oxygen vacancies. The W_18_O_49_ NW film also shows a moderate coloration efficiency (CE) of 185.0 cm^2^ C^−1^ at 1200 nm and 70.5 cm^2^ C^−1^ at 633 nm (Fig. 2f).

The detail electrochemical behaviors of Li^+^ in W_18_O_49_ NW film were characterized by cyclic voltammetry (CV) measurements at different scan rates as shown in the Fig. 2g. The CV curves show no prominent redox peaks that are commonly associated with the phase transition. During the cathodic scanning from 1 to − 0.2 V, the Li^+^ mainly absorb on the surface of W_18_O_49_ NWs, while the charge-compensating electrons are injected in W_18_O_49_ NWs that gives rise to the electrochemical tuning of localized surface plasmon resonance (LSPR) in the NIR region. When the potential is below − 0.2 V, the current increases sharply which indicates that the Li^+^ intercalate into the W_18_O_49_ NWs resulting in polaron absorption in the VIS range. The surface adsorption assisted LSPR tuning mechanism was also certificated by using a large size of bulky cation (tributylmethylammonium, TMA^+^) which cannot intercalate into the W_18_O_49_. The NW structure endow it high surface area for ion absorption. Figure 2h shows that W_18_O_49_ NW film can still selectively modulate NIR by capacitive charging (surface absorption), which is similar to the case of the previous reported plasmonic semiconductors. Consequently, the electrochromic modulation of NIR and VIS light can be attributed to LSPR and polaron absorption respectively. The W_18_O_49_ NWs also show a fast diffusion coefficient of 1.39 × 10^–10^ cm^2^ s^−1^ (Fig. 2i), which is calculated by the Randles-Sevick equation from the voltammograms measured at different scan rates. Ex situ XRD (Fig. 2j) measurements also indicated no phase transformations during the Li^+^ absorption and intercalation process, which is expected to improve the cycle stability. The electrochemical energy storage performance of W_18_O_49_ NW film is shown in Fig. 2k, m. The galvanostatic charge/discharge (GCD) performance of the film displays the relatively symmetric curves, indicating a good reversibility of Li^+^ ion insertion/extraction. The W_18_O_49_ NW electrode exhibits high areal capacitances of 147, 125.4, 110.8, and 105 F m^−2^ at the current densities of 0.2, 0.3, 0.4, and 0.5 mA cm^−2^, respectively, suggesting a good rate performance (Fig. 2m).

### Electrochromic Properties of the Flexible DBED

**Fig. 3:**
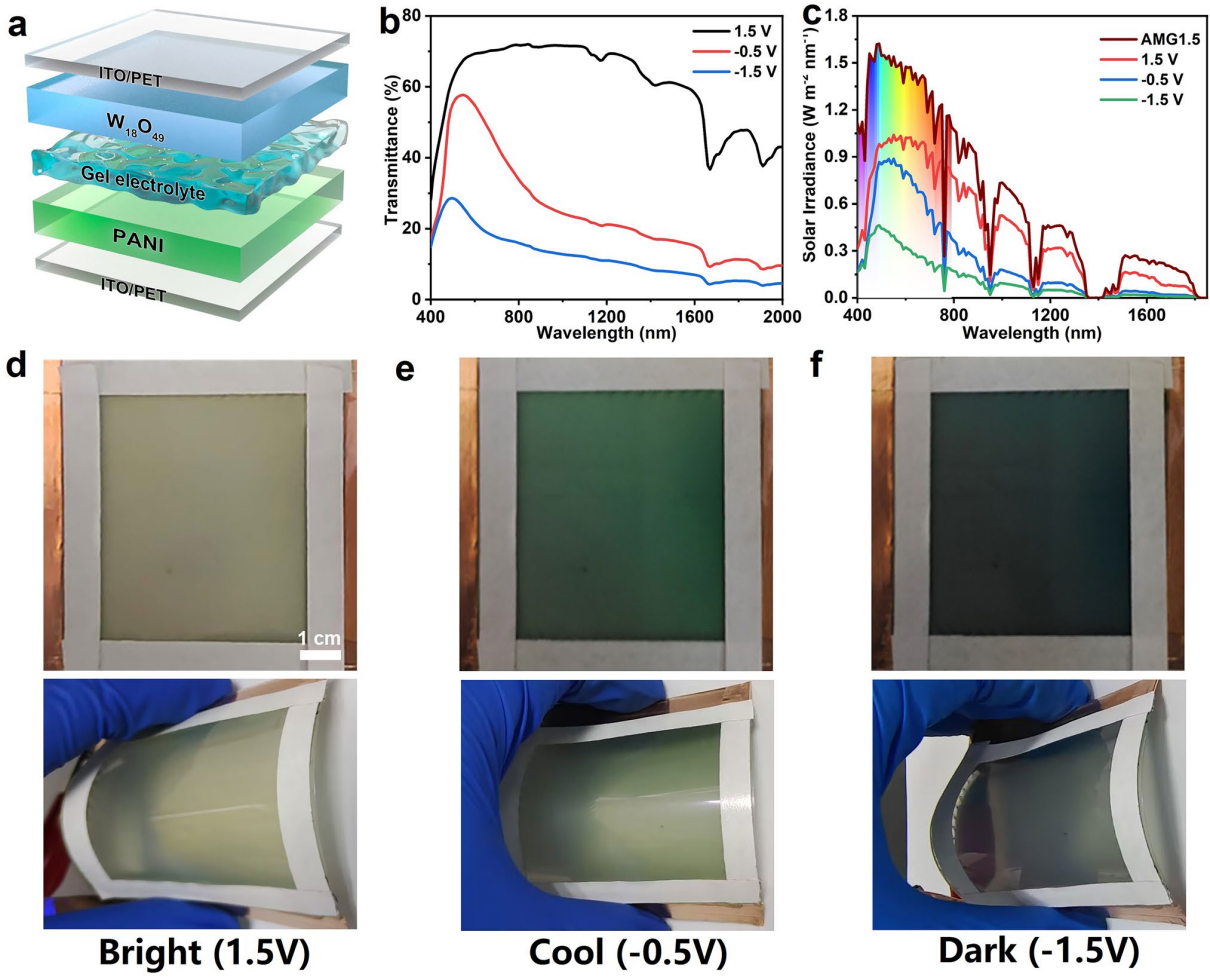
**a** Schematic diagram of the flexible DBED based on a W_18_O_49_ NW cathode and a PANI anode. **b** Optical transmittance spectra, **c** solar irradiance spectra and **d-f** corresponding digital photos of DBED (size: 7 × 7 cm^2^) in the bright (1.5 V), cool (− 0.5 V), dark (− 1.5 V) modes, respectively

The application performance of W_18_O_49_ NW film was also evaluated at the full cell level using a prototype flexible dual-band electrochromic device (DBED) consisting of a W_18_O_49_ NW cathode and a porous polyaniline (PANI) anode in LiClO_4_/PC gel electrolyte (Fig. 3a). The flexible PANI anodes prepared by electrodeposition show a porous structure (Fig. S5) which is beneficial to the ion fast transport, and also show good electrochromic performance with a moderate optical modulation, fast response speed and high coloration efficiency (Fig. S6). The porous PANI anodes not only can provide an ion storage function to balance the cathode charge, but also are able to enhance the optical modulation performance of the device. Due to the reasonable design of the device’s structure, the operating voltage of DBED was reduced to 1.5 V which is smaller than most of inorganic electrochromic devices (usually require 2–3 V) [28–39], contributing to a lower energy consumption of the device itself. Figure 3b shows that the DBED delivers as good dual-band electrochromic performance as in the three-electrode measurements, modulating the NIR and VIS light independently and efficiently through the three modes with a high optical modulation of 51.7% at 633 nm and 60.0% at 1,200 nm. In the cool mode (-0.5 V), the DBED is able to block 76.3% of solar heat in the NIR range for building heat load reduction while providing a high VIS light transmittance of 46.4% for daylighting (Fig. 3c and Table 2). Figure 3d-f shows the corresponding digital photos of the flexible DBED in bright, cool and dark modes. The DBED can still work normally in the bent states, demonstrating the feasibility of our design.

**Table 2:** Integrated optical transmittance (T) and integrated solar irradiance transmittance (T′) of DFED in the VIS light (400–780 nm), NIR (780–2000 nm); and total solar irradiation (sol, 400–2000 nm) at 1.5, -0.5 and -1.5 V

Mode	T_VIS_ (%)	T_NIR_ (%)	Tsol (%)	T_VIS_′ (%)	T_NIR_′ (%)	Tsol′ (%)
Bright (1.5 V)	63.6	59.8	60.7	63.7	67.4	65.3
Cool (− 0.5 V)	45.2	18.3	24.7	46.4	23.7	36.5
Dark (− 1.5 V)	23.7	8.5	12.1	24.4	11.2	18.6

The calculations are based on the following equations: $\text{Tsol}=\frac{\int T\left(\lambda \right)d\lambda }{\int d\lambda }$, ${\text{Tsol}}^{\prime }  = \frac{{\int {\text{T}} \left( \lambda  \right)\psi (\lambda ){\text{d}}\lambda }}{{\int \psi  (\lambda ){\text{d}}\lambda }}$, where $T(\lambda )$ is the transmittance at wavelength of $\lambda$, and $\psi (\lambda )$ is the solar irradiance at 1.5 air mass

**Fig. 4:**
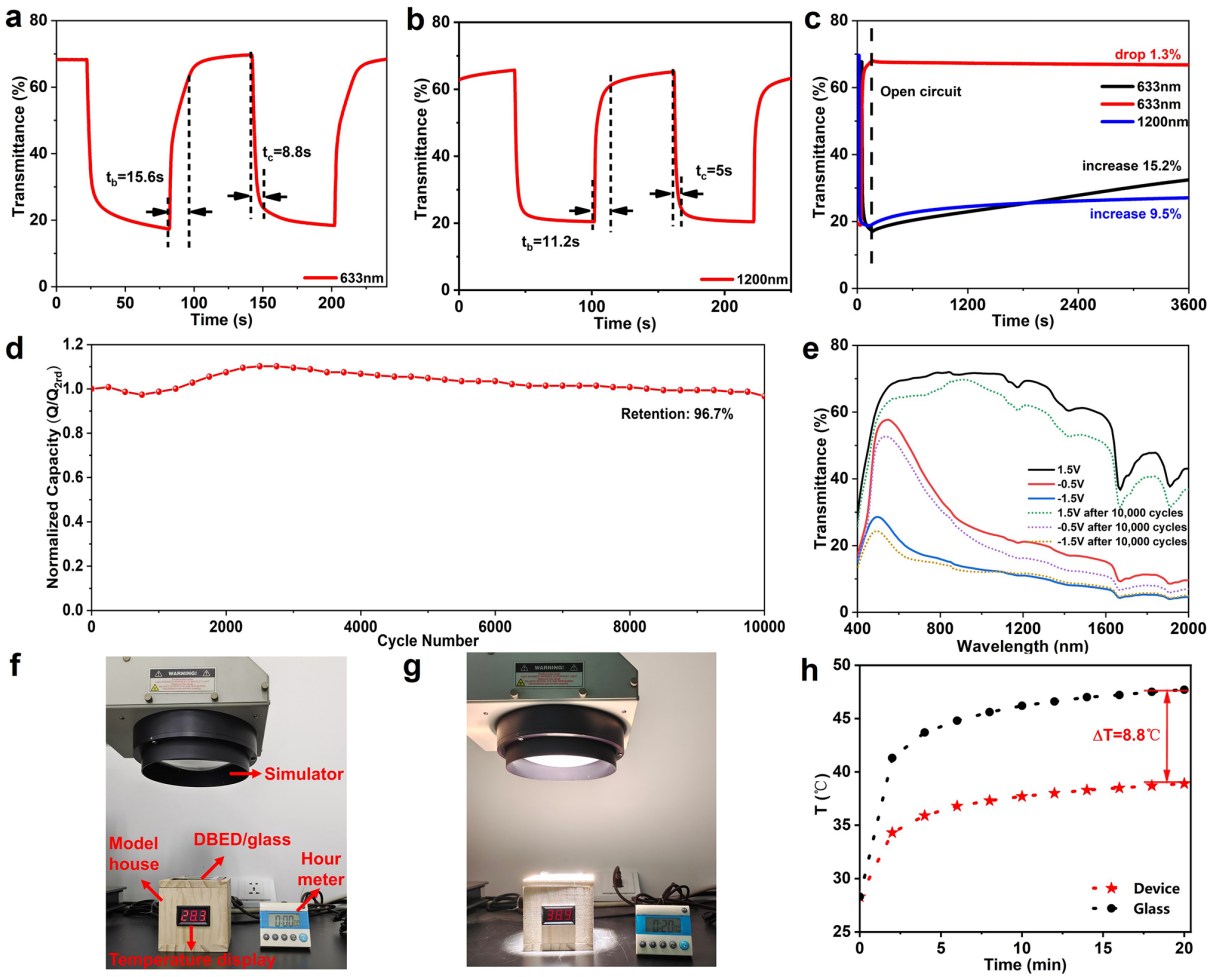
**a** Real-time transmittance spectra of DBED at 633 nm (− 1.5 to 1.5 V) and **b** 1200 nm (− 0.5 to 1.5 V). **c** Transmittance changes at 633 and 1200 nm under the open-circuit condition after the DBED was applied with potential of 1.5, − 0.5 and − 1.5 V for 60 s. **d** The normalized capacity of DBED over 10,000 galvanostatic charge/discharge cycles between − 1.5 and 1.5 V at 1 mA cm^−2^. **e** Optical transmittance spectra of DBED before and after 10,000 cycles. **f** The digital photos of the model room with DBED before and **g** after espousing to simulated sunlight for 20 min. **h** Temperature changes in the model room for installing DBED and common glass

Except the optical modulation, the switching speed, bistability, cycle stability and energy-saving performance of the DBED were also investigated detailly in our study. Figure 4a, b shows that the DBED delivers fast switching speeds with *t*_c_/*t*_b_ of 8.8/15.6 s and 5.0/11.2 s at 633 and 1200 nm respectively, yielding a moderate CE of 52.6 and 127.9 cm^2^ C^−1^ at 633 and 1200 nm respectively (Fig. S7). The electrochemical impedance spectroscopy (EIS) was conducted to characterize the contact resistance (*R*_s_) and charge transfer resistance (*R*_ct_) of dual-band electrochromic device. As shown in Fig. S8, the *R*_s_ and *R*_ct_ are 131.5 and 54.9 Ω respectively. The relatively large contact resistance can be attributed to the relatively low conductivity of the W_18_O_49_ NW film. The low charge transfer resistance and the high slope in the low-frequency region of the EIS plot indicate that the device has a low ion diffusion resistance. To quantify the capacitive contribution, we used the formula: i = k_1_ν + k_2_ν^1/2^, where i (A) is the peak currents, v (mV s^−1^) is the scan rates (ν), k_1_ and k_2_ are proportional to the capacitance and diffusion contribution, respectively, to calculate the total surface capacitance and ion diffusion contribution rate of the device. As depicted in Fig. S9, the capacitive contribution rate increases from 25.5% to 53.3% as the scan rate increases from 2 to 30 mV s^−1^, demonstrating increasing dominance of surface capacitive effects, which is responsible for the device's rapid electrochemical response. The video (Supplementary file2) of the DBED in the color-switching process also demonstrates the good electrochromic performance. Bistability (optical memory) is an important indicator of electrochromic properties for smart windows. It refers to the ability to maintain the colored or bleached state under the open-circuit condition. Figure 4c shows good bistability of DBED in the three operating modes. After the application of an external voltage (1.5, − 0.5, and − 1.5 V) to turn on the desired states for 60 s and then open-circuited, the transmittance of the bright (633 nm), cool (1200 nm) and dark (633 nm) states varies by 1.3%, 9.5%, and 15.2%, respectively, after 3600 s. Remarkably, the DBED exhibits an excellent cycling stability with 96.7% of capacity retention (Fig. 4d) and 93.3% of optical retention (Fig. S11) after 10,000 cycles, which is better than most of the reported DBEDs (Table S1)*.* As shown in Fig. S11, the nanowire morphology of W_18_O_49_ NWs was still maintained even after 10,000 cycles, which further confirmed their outstanding cycling stability. Figure 4e shows that the device still can modulate the VIS and NIR selectively with a high optical modulation after 10,000 cycles, which can be attributed to the high structure stability of W_18_O_49_ NWs during the Li^+^ intercalation/de-intercalation process. In order to further demonstrate the energy-saving performance of the device, we establish an apparatus as illustrated in Fig. 4f to simulate the modulation of the room temperature with the assembled DBED. The model house with the size of 10 × 10 × 10 cm^3^ has a hole of 5 × 5 cm^2^ at the top, which can be covered by DBED or common glass. Under the stimulated sunlight (AM 1.5, 1000 W m^−2^) for 20 min, the interior temperature of the room installed with DBED (in the dark state) is raised from 28.3 to 38.9 °C (Fig. 4f, g), while the interior temperature of the room installed with common glass (Fig. S12) is increased from 28.2 to 47.7 °C (Fig. 4h). The DBED exhibits 8.8 °C lower than the common glass, thus the energy consumption especially on air-conditioning can be notably reduced.

### Thermal Modelling and Energy-Saving Evaluation

**Fig. 5:**
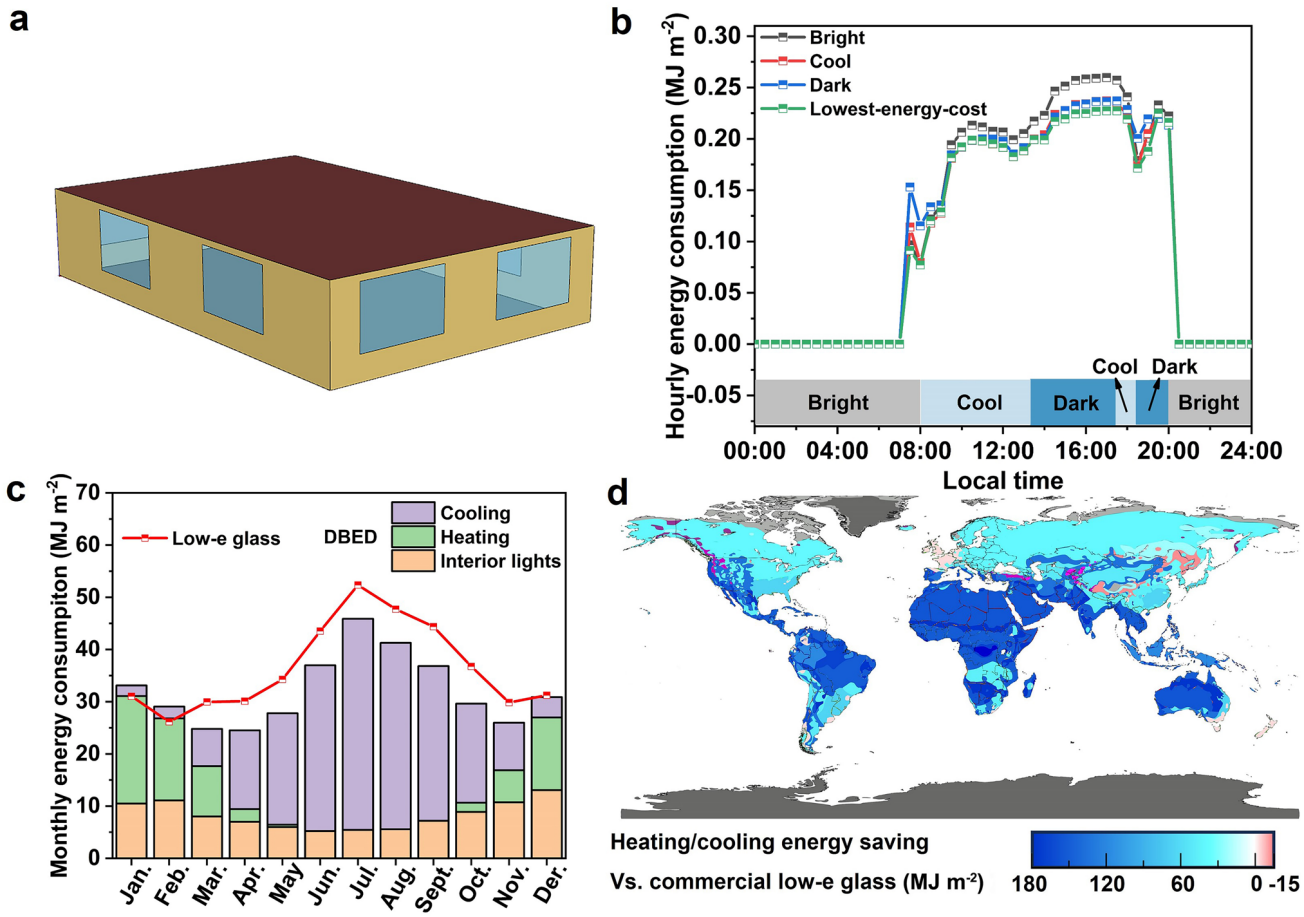
**a** Small office prototype building model in EnergyPlus. **b** Hourly energy consumption of DBED in bright, cool, dark and lowest energy cost states in the climate condition of Nanjing in July. The working state of DBED in lowest energy cost state as follows: 00:00–8:00 and 20:00–24;00, bright; 08:00–13:30 and 17:30–18:30, cool; 13:30–17:30 and 18:30–20:00, dark. **c** Monthly energy consumption of DBED and low-e glass in the climate condition of Nanjing. **d** Estimated heating and cooling energy saving of the DBED against a commercial low-e glass as the baseline for the different climate zones across the world

In order to further explore the energy-saving performance of the DBED, we performed extensive simulations based on the device properties and the climate database. Here we used EnergyPlus to evaluate the heating, ventilation, and air conditioning (HVAC), along with the lighting energy-saving performance. A full-scale energy simulation was conducted on an office prototype building model with the dimensions of 15 × 10 × 3 m^3^ and eight 3 × 2 m^2^ windows (Fig. 5a and Table S2). For a more precise fit with actual needs, indoor air temperature is set to fluctuate according to the time of day as shown in the Fig. S13. To comprehensively explore the energy-saving capabilities of DBED, we have also simulated the lighting system. In our simulation, the indoor lighting is set to zero power consumption at 0 W m^−2^ from 8:00 PM to 7:00 AM. During the daytime, the system aims to maintain an illuminance of 500 lx. When the ambient light exceeds this level, the lighting power is 0. When the ambient illuminance is lower than 500 lx, the artificial lighting system is activated with power output increasing linearly from 0 to a maximum of 8 W m^−2^ to ensure the desired illuminance (Fig. S14). To determine the most optimal energy-efficient state of the DBED under various weather conditions, we conducted a comprehensive comparison of the energy consumption of the three distinct optical states around the world across different climate zones. From these comparisons, the optical state with the lowest energy cost was selected as the finial state, which was then incorporated into the annual energy statistics to reflect the most energy-efficient scenario throughout the year. Nanjing is choosed as a model city to illustrate the selection strategy of the optical states in response to different ambient temperatures and solar illuminance. Figure 5b illustrates that the DBEDs with dynamic switching among three distinct states in various scenarios show the lowest energy consumption on July of Nanjing, outperforming the static maintenance of a single state. The bright mode allows the VIS light and NIR into the buildings for lighting and heating under low temperature. As the temperature increases, the DBED switches into cool mode with high VIS transmittance and low NIR transmittance to minimize the cooling and lighting energy consumption. Under high temperature, the dark mode is activated to block most of solar irradiances to further reduce the cooling demands.

Additionally, we also conducted the monthly energy consumption of the optimized DBED and commercial low-emissivity (low-e) glass in Nanjing to evaluate energy-saving potential of the device. As shown in Fig. 5c (more details about the parameters of windows are given in Table S3), the optimized DBED displays the lower energy consumption in the whole year than the commercial low-e glass. The DBED can save 11.6% of the annual energy consumption comparedwith low-e glass (Fig. S15), demonstrating its high energy-saving potential in the practical applications. In order to comprehensively evaluate the adaptability and energy-saving performance of the DBED across different climate zones, we also conducted the simulations in different climate zones around the world. Our optimized DBED yielded a higher energy-saving performance than the commercial low-e glass across almost all different climate conditions (Fig. 5d), with energy saving up to 178.3 MJ m^−2^ (Phoenix), further demonstrating the potential of DBED for energy-efficient buildings. More detailed information about energy-saving performance simulation is given in Table S4.

### Energy Storage and Energy Recycling Performance of DBED

**Fig. 6:**
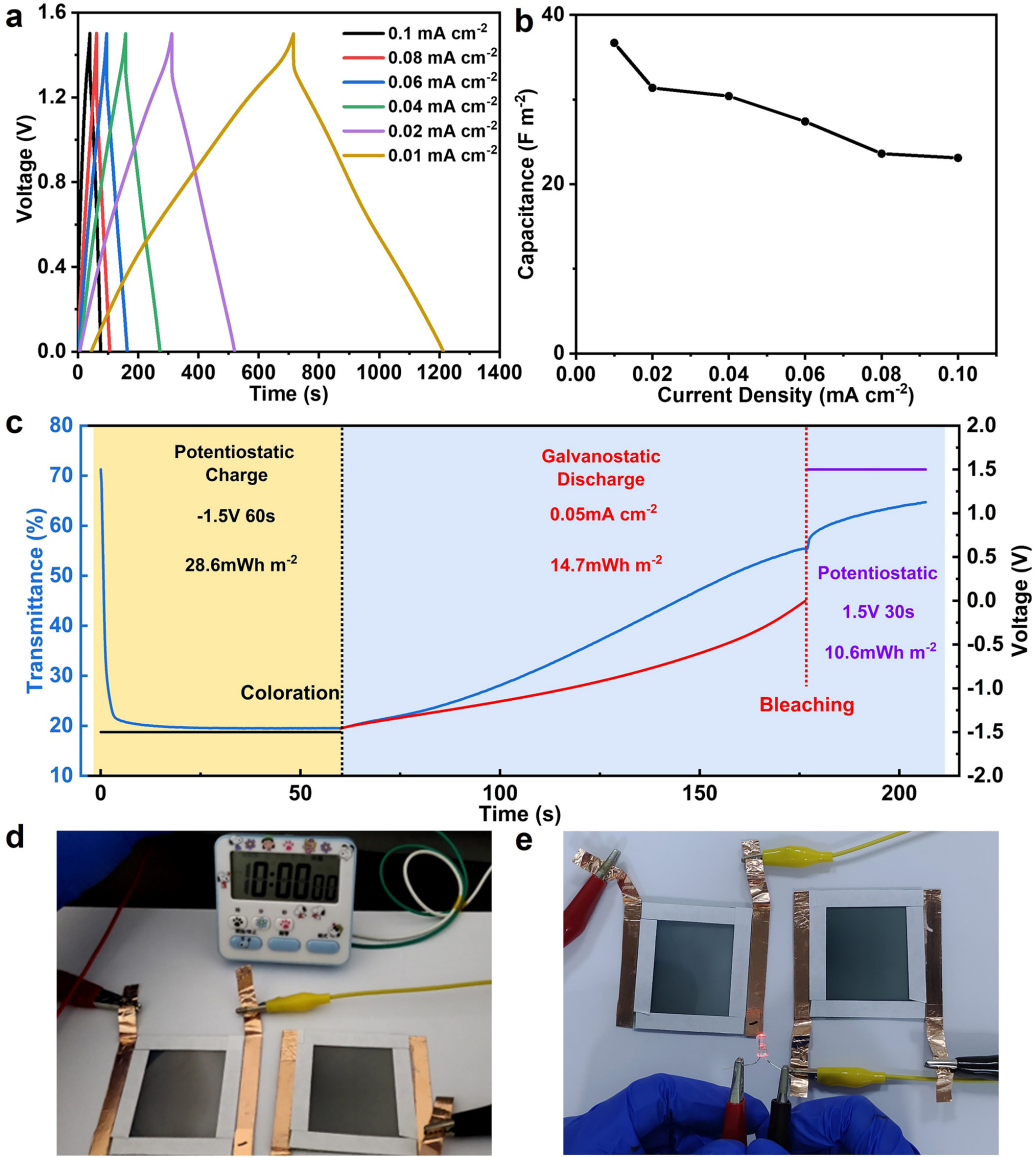
**a** The galvanostatic charge/discharge curves and **b** areal capacitance of the DBED at different current densities. **c** Potentiostatic charge curve (black line) at − 1.5 V for 60 s, galvanostatic discharge curve (red line) at a current density of 0.05 mA m^−2^; potentiostatic charge curve (purple line) at 1.5 V for 30 s; and the corresponding changes of the transmittance at 633 nm measured in situ (blue line). **d** The digital photos of electronic clock and **e** LED powered by two DBEDs (size: 5 × 4 cm^2^)

Due to the similar device structure and operating principle with rechargeable batteries [41], the DBEDs also possess an energy storage function. The electrical energy consumed in the coloration process can be transformed into chemical energy and stored within the device, which can be released in the opposite electrochromic operation for local reuse or uploaded to a connected grid (“energy recycling”). The net energy consumption of the device in a round-trip electrochromic operation can be substantially reduced if there is efficient energy recycling. Hence, the energy storage performance and the net energy consumption of the flexible DBED in a round-trip coloration and bleaching operation were analyzed in detail in this study. As shown in Fig. 6a, the DBED exhibits a good energy storage performance with areal capacitances of 36.7, 31.4, 30.4, 27.4, 23.6, and 23.1 F m^−2^ at the current densities of 0.01, 0.02, 0.04, 0.06, 0.08, and 0.1 mA cm^−2^ respectively, suggesting a good rate performance (Fig. 6b). The energy recycling performance of the DBED was evaluated detailly in a round-trip electrochromic operation. During coloration, the DBED was charged potentiostatically at − 1.5 V for 60 s, consuming 28.6 mWh m^−2^ of energy (Fig. 6c), while the transmittance of the device decreased from 71.3% to 19.6%. In the bleaching process, the DBED was first discharged to 0 V at a high current density of 0.05 mA cm^−2^, releasing 14.7 mWh m^−2^ of energy in this process (Fig. 6c). Therefore, the energy recycling efficiency (energy released in the discharge process/energy consumed in the coloration process) of DBED is as high as 51.4%. More than half of the energy consumed in the coloration process can be recycled and reused, which can notably reduce the net energy consumption of the device. However, the DBED could not return to its fully transparent state by this discharge process. A positive voltage (1.5 V) was needed for 30 s to fully extract the Li^+^ from the lithiated W_18_O_49_ and return the device to the fully transparent state, consuming an additional 10.6 mWh m^−2^ of energy (Fig. 6c). The net energy consumption (energy consumed—energy released) of the DBED in an overall coloration and bleaching processes was therefore reduced to 24.5 mWh m^−2^ (28.6 + 10.6 – 14.7 = 24.5) due to efficient energy recycling and partial self-bleaching, which is lower than most of electrochromic devices (usually > 50 mWh m^−2^) [28–39]. For demonstrating the energy recycling performance of DBED, the energy released in the discharge process can be used to power the electronic clock (Fig. 6d) or light up the red LED (Fig. 6e).

## Conclusions

In conclusion, this study presents an efficient and flexible bifunctional dual-band electrochromic device with a high optical modulation, a long cycle life, a high capacitance and energy recycling efficiency, integrating energy-saving with energy-storage. The nanowire structure, abundant oxygen-vacancies and high surface area of W_18_O_49_ NWs endow it high flexibility, tunable LSPR and adequate ion storage sites. Consequently, the W_18_O_49_ NW film delivers impressive dual-band electrochromic performance including high optical modulation, high spectral-selectivity and fast switching speed. The dual-band mechanism can be illustrated by the electrochemically tunable LSPR (Li^+^ adsorption/desorption) and polaron absorption (Li^+^ intercalation/de-intercalation). The assembled prototype flexible device is able to modulate the VIS and NIR independently and effectively through three distinct modes with high optical modulation, fast response speed, and long cycle life (3.3% capacity loss after 10,000 cycles). The cycling stability is significantly improved due to the high structure stability of monoclinic W_18_O_49_ during Li^+^ intercalation/de-intercalation without any phase transition. The practical energy-saving performance of the device is experimentally demonstrated in this work, showing 8.8 °C lower than the common glass under simulated sunshine. Furthermore, simulations also demonstrate that the device exhibits higher energy-saving performance than the commercial low-e glass in most climatic zones around the world. The flexible device also delivers good energy storage and energy recycling performances. 51.4% the energy consumed in the coloration process can be recycled and reused, thus the net energy consumption of the device in a round-trip electrochromic operation is reduced to only 24.5 mWh m^−2^. The excellent spectral-selective modulation and efficient energy recycling notably reduce the energy consumption of the buildings and the electrochromic devices. This study not only confirms the capability of W_18_O_49_ NWs as a single-component flexible dual-band electrochromic material, but also address the issue of low optical modulation and poor durability of current dual-band electrochromic devices, providing the rational approach to the design of flexible bifunctional dual-band electrochromic devices for energy-efficient buildings.

## Supplementary Information

Below is the link to the electronic supplementary material.

Supplementary file1 (MP4 4556 kb)

Supplementary file2 (DOCX 3253 kb)
