# Supplementary material for: An Efficient and Flexible Bifunctional Dual-Band Electrochromic Device Integrating with Energy Storage
Source: Nanomicro Lett. 2024 Dec 27;17:98. doi: 10.1007/s40820-024-01604-0 (PMC11680540; doi:10.1007/s40820-024-01604-0)
Supplement: Supplementary file 2 — Supplementary file2 (DOCX 3253 kb) [file 40820_2024_1604_MOESM2_ESM.docx]

Supporting Information for

**An Efficient and Flexible Bifunctional Dual-Band Electrochromic Device Integrating with Energy Storage**

Zekun Huang^1,#^, Yutao Peng^1,#^, Jing Zhao^1,#^, Shengliang Zhang^1,2,^*, Penglu Qi^1^, Xianlin Qu^3^, Fuqiang Yan^1^, Bing Ding^1^, Yimin Xuan^2,^* and Xiaogang Zhang^1,^*

^1^ Jiangsu Key Laboratory of Electrochemical Energy Storage Technologies, College of Materials Science and Technology, Nanjing University of Aeronautics and Astronautics, Nanjing 210016, P. R. China

^2^ Key Laboratory of Thermal Management and Energy Utilization of Aviation Vehicles, College of Energy and Power Engineering, Nanjing University of Aeronautics and Astronautics, Nanjing 210016, P. R. China

^3^ Center for Microscopy and Analysis, Nanjing University of Aeronautics and Astronautics, Nanjing 211106, P. R. China

^#^ Zekun Huang, Yutao Peng, and Jing Zhao contributed equally to this work.

*Corresponding authors. E-mail: [zhangsl@nuaa.edu.cn](mailto:zhangsl@nuaa.edu.cn) (Shengliang Zhang); [ymxuan@nuaa.edu.cn](mailto:ymxuan@nuaa.edu.cn) (Yimin Xuan); [azhangxg@nuaa.edu.cn](mailto:azhangxg@nuaa.edu.cn) (Xiaogang Zhang)

Supplementary Equation, Figures and Tables

The solvothermal reaction of WCl_6_ with C_2_H_5_OH produces W_18_O_49_, which can be represented by the chemical equation S1:

WCl_6_ + 2C_2_H_5_OH → W_18_O_49_ + 6HCl + 2CH_3_CHO (S1)


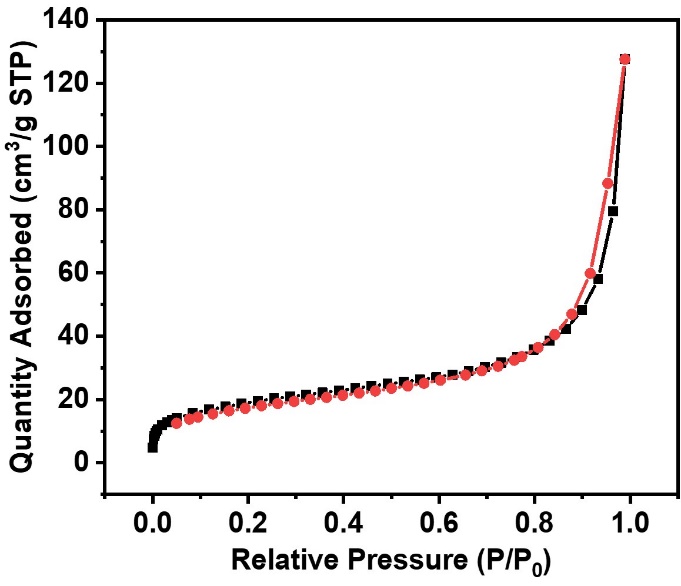


**Fig. S1** Nitrogen adsorption/desorption isotherms of W_18_O_49_ NWs


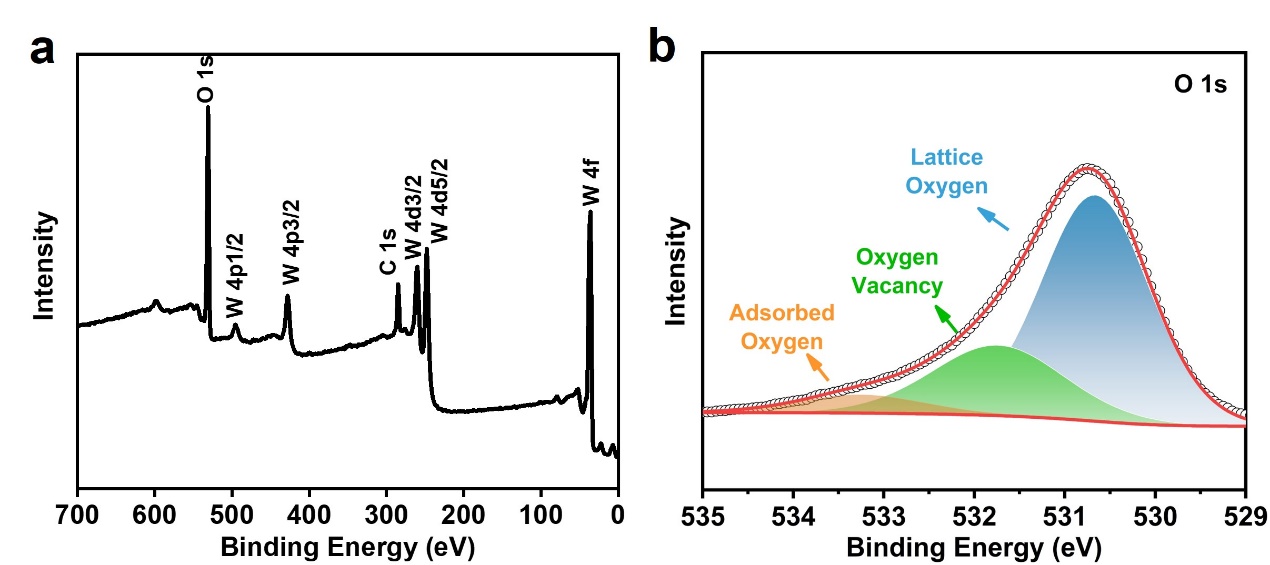


**Fig. S2** XPS survey spectra of **a** W_18_O_49_ NWs and **b** O 1s

**
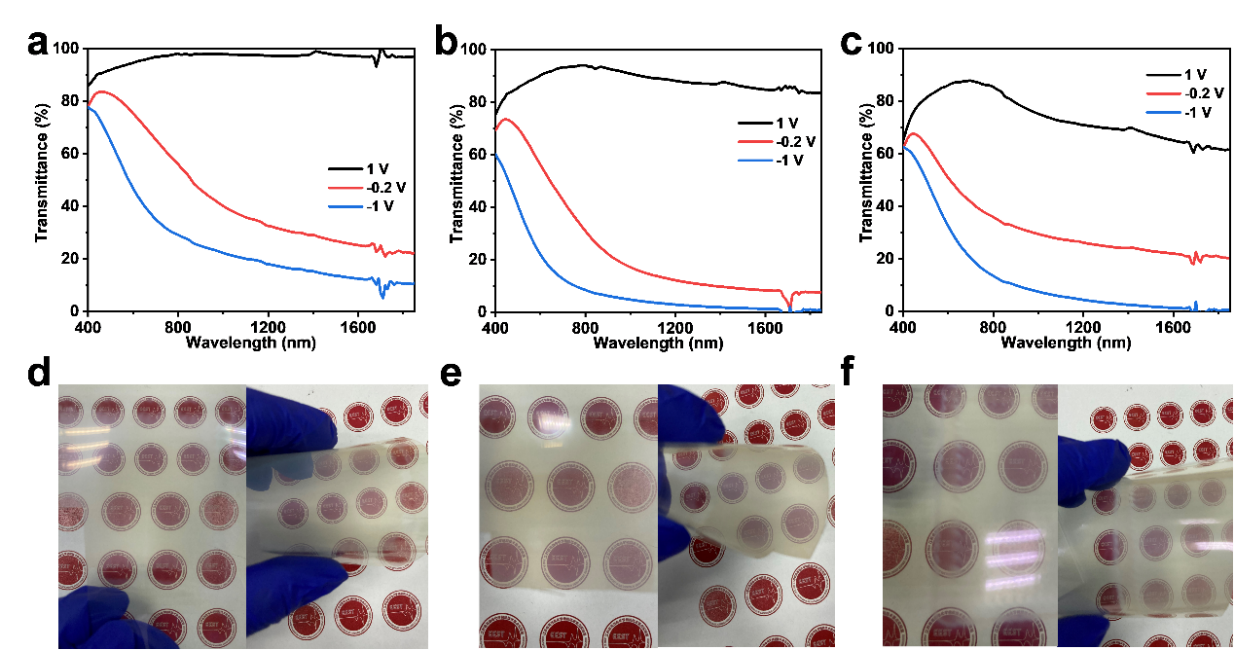
**

**Fig. S3** The transmittance spectra of W_18_O_49_ NWs films with different ultrasonic spraying times. **a** 50, **b** 100, **c** 150. The digital photo of W_18_O_49_ NWs films with different ultrasonic spraying times. **d** 50, **e** 100, **f** 150

**
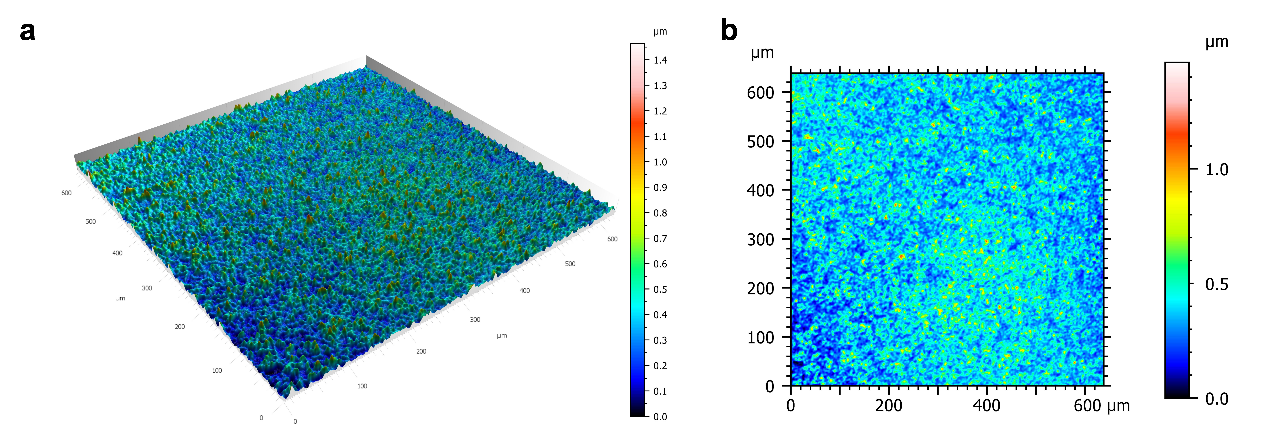
**

**Fig. S4** laser confocal microscopy Surface roughness of W_18_O_49_ NWs film sprayed 100 times

**
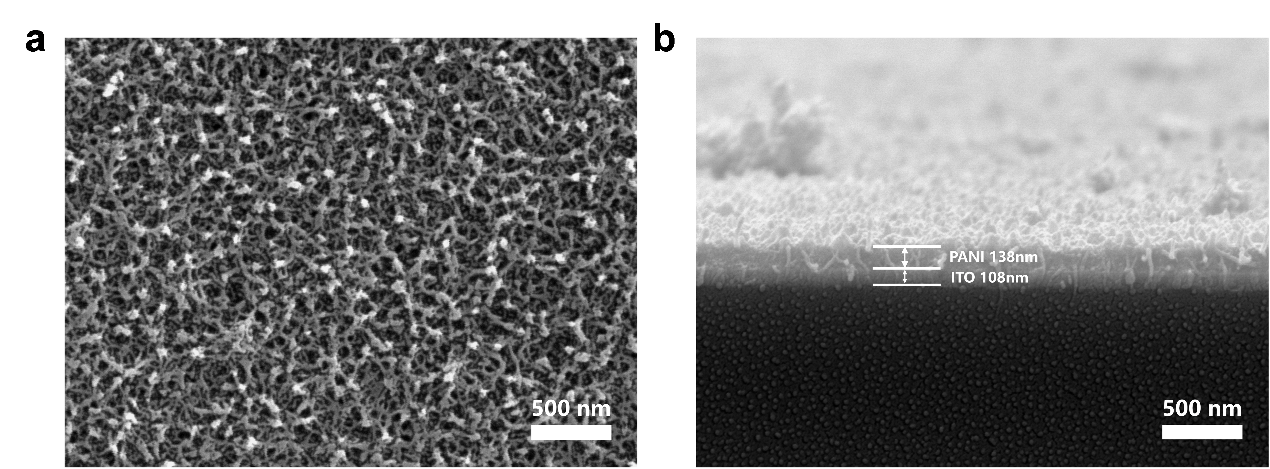
**

**Fig. S5** **a** Surface SEM image of porous PANI anodes. **b** Cross-sectional SEM image of porous PANI anodes


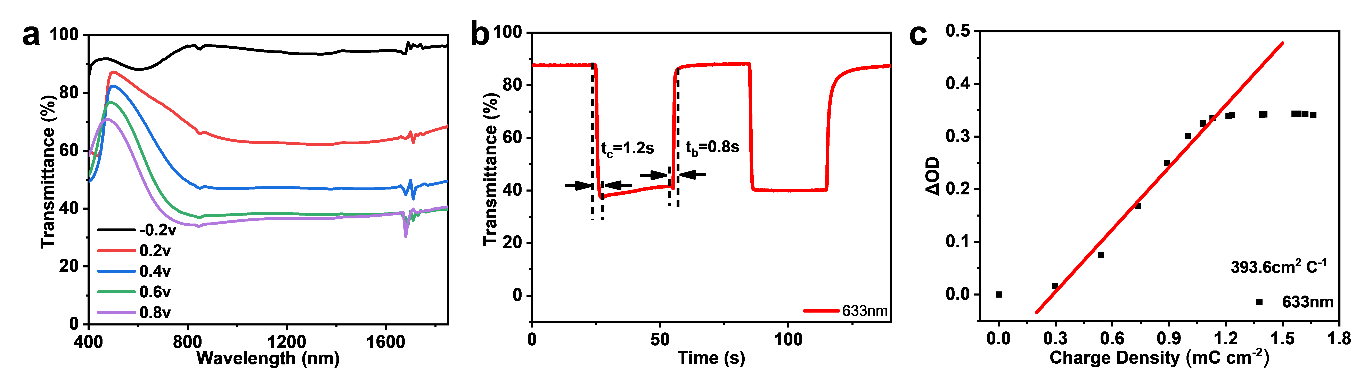


**Fig. S6** **a** Transmittance spectra of PANI anodes at different applied potentials. **b** Real-time transmittance spectra at 633 nm of PANI anodes. **c** Optical density changes of PANI thin films at 633 nm as a function of injected charge density


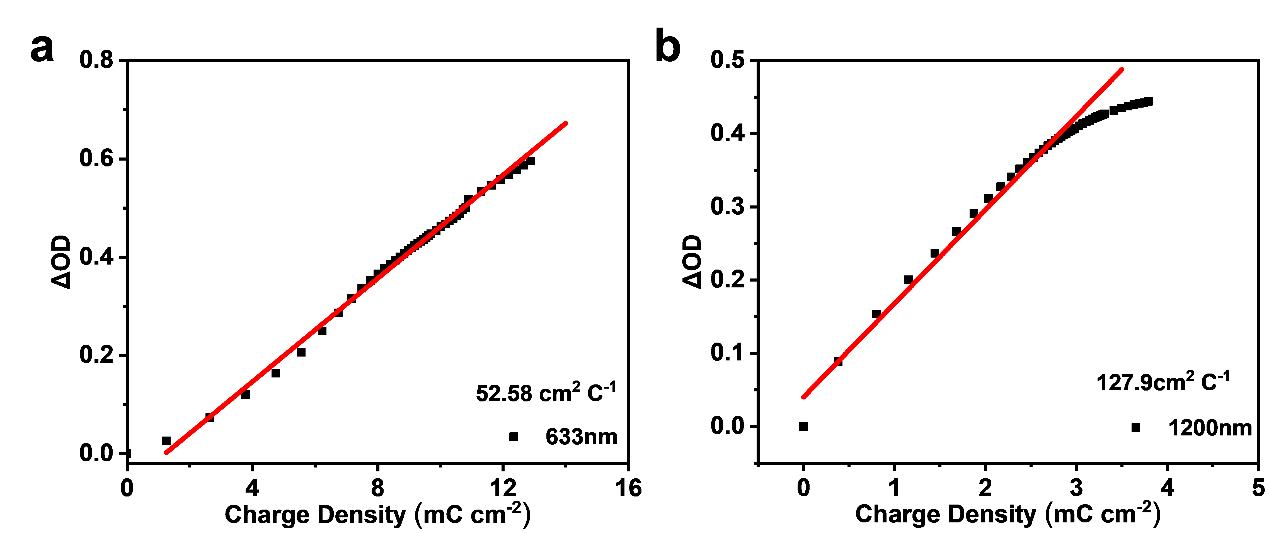


**Fig. S7** Optical density changes of DBED at **a** 633 and **b** 1200 nm as a function of injected charge density


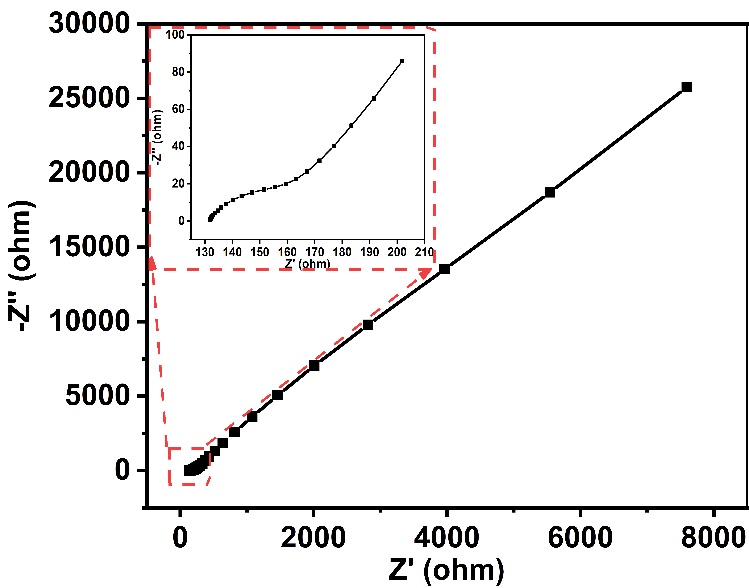


**Fig. S8** Impedance spectra of the DBED. Inset shows the enlarged impedance curve


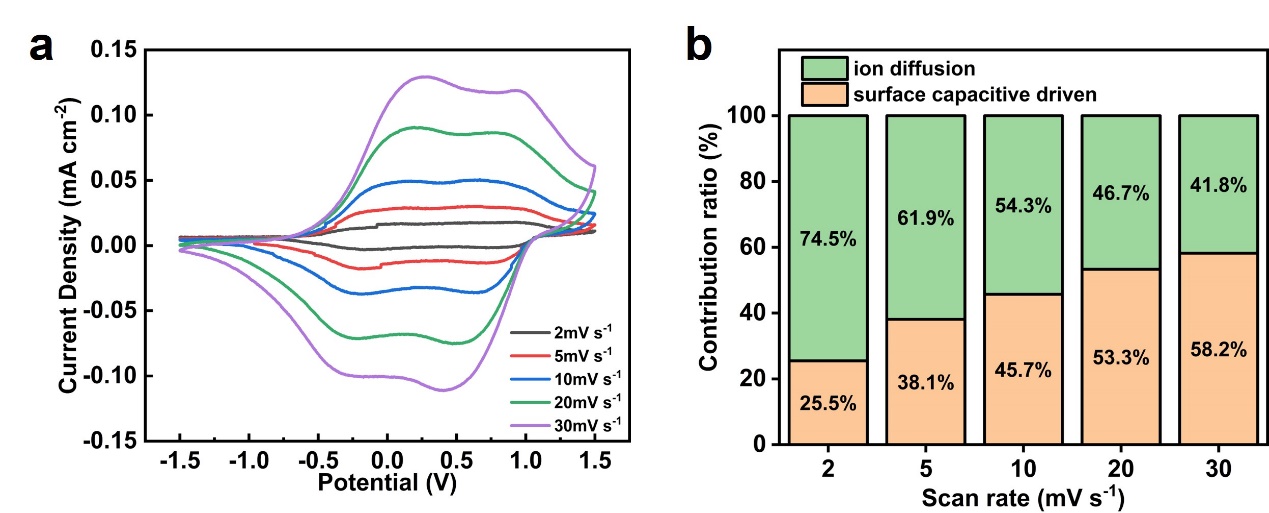


**Fig. S9 a** Cyclic voltammograms and **b** the ratios of capacitance and diffusion-controlled contribution of DBED at different scan rates


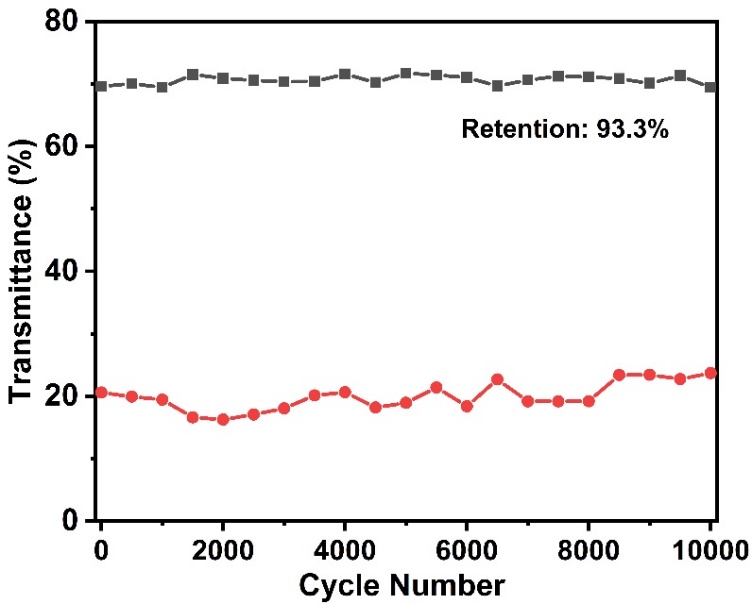


**Fig. S10** Optical transmittance changes (at 633 nm) of DBED over 10,000 cycles between 1.5 and −1.5 V for 30 s at each potential


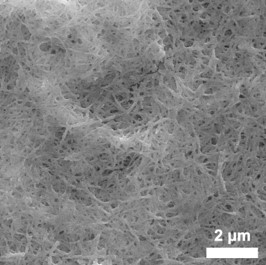


**Fig. S11** Surface SEM images of W_18_O_49_ NW film after cycling


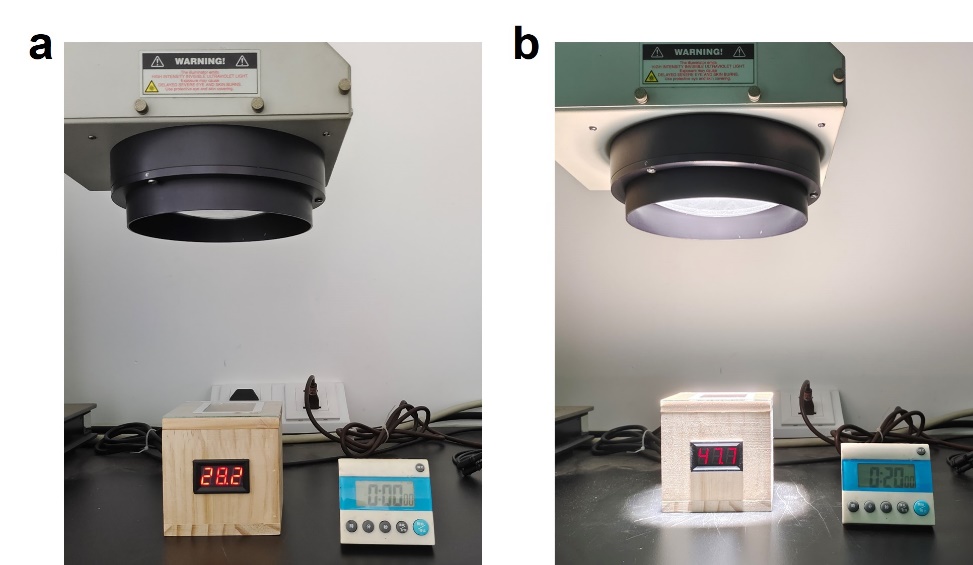


**Fig. S12** The digital photos of the model room with common glass **a** before and **b** after espousing to simulated sunlight for 20 minutes


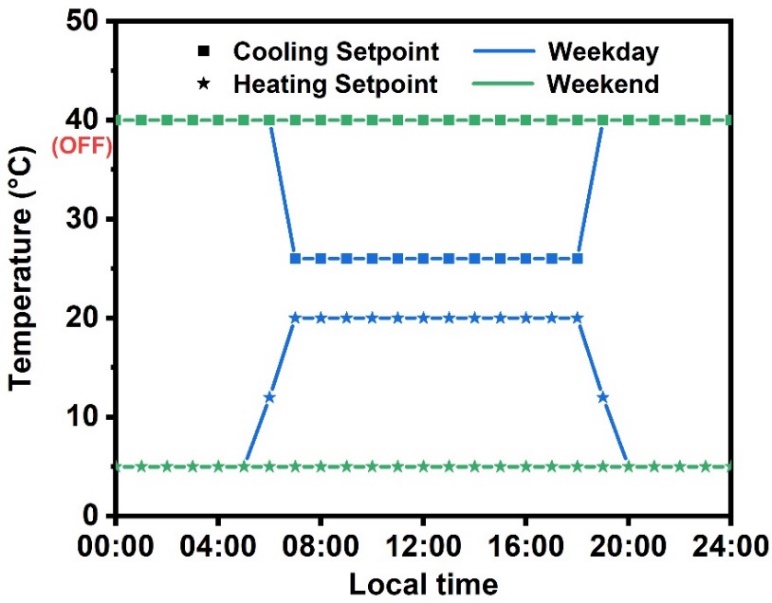


**Fig. S13** The thermostat setpoints applied in actual building energy simulation


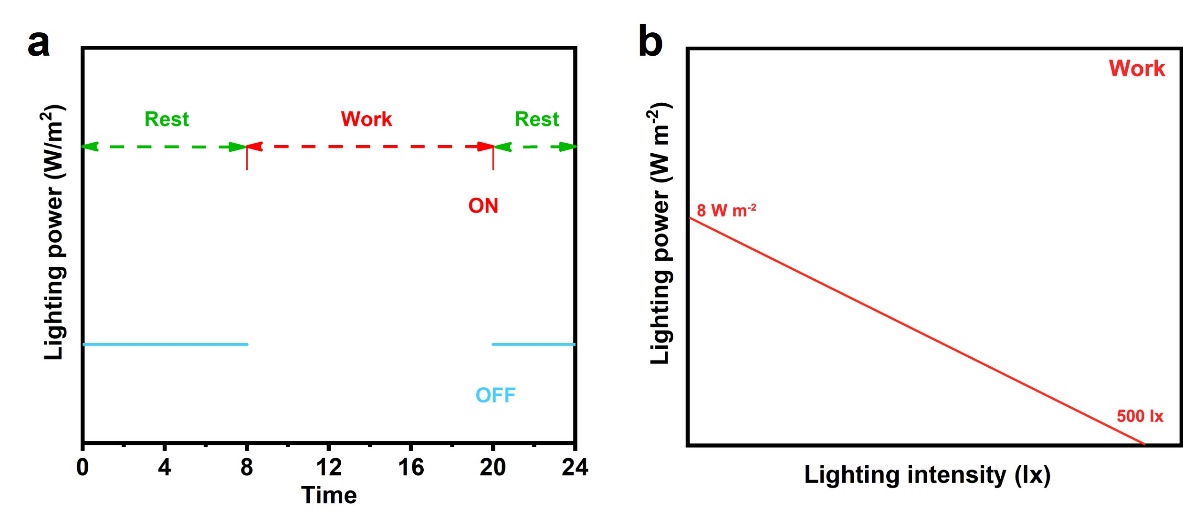


**Fig. S14** **a** Lighting set for the simulation of lighting environment of human activity. During the night from 20:00 to 8:00, the interior light was set constantly as 0 W m^-2^. During the daytime, the illuminance setting value is 500 lx. **b** When the daytime illuminance is higher than 500 lx, the lighting power is 0. When the daytime illuminance is lower than 500 lx, the lighting power will increase linearly from 0 to 8 W m^-2^


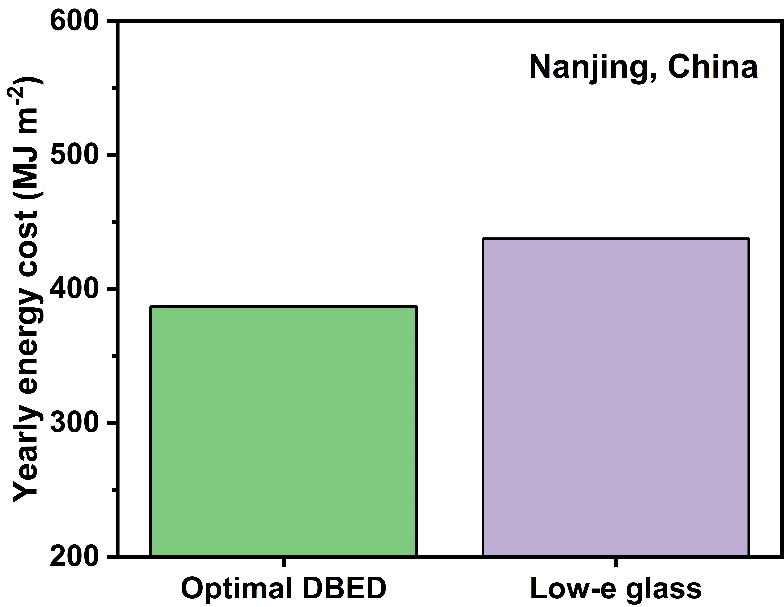


**Fig. S15** Energy cost of optimal DBED, and commercial low-e glass in the climate condition of Nanjing

**Table S1** Comparison of electrochromic performance of currently available dual-band electrochromic materials. (△T_633nm_ and △T_1200nm_ are the optical modulation at 633nm and 1200 nm respectively, t_c_ and t_b_ are respectively, the coloration time and the bleaching time. CE is coloration efficiency. N/A indicates data is not available.

| **Material** | **△T_633nm_** | | | **△T_1200nm_** | **t_c_/t_b_ (s)** | **CE (cm^2^ C^-1^)** | **Cycle stability** |
| --- | --- | --- | --- | --- | --- | --- | --- |
| ITO/NbO_x_ [S1] | | ~35% | | ~17% | N/A | 30 ± 4 (500 nm） | 2000 cycles  (4% capacity loss) |
| WO_3-x_/NbO_x_ [S2] | | ~80% | | ~85% | 600/100  (633 nm) | N/A | 2000 cycles  (5.7% capacity loss) |
| W_18_O_49_/PB [S3] | | | 71.2% | ~50% | 2.4/4.2  (633 nm) | N/A | 100 cycles  (30% capacity loss) |
| P_8_W_48_/W_18_O_49_ [S4] | | | ~75% | ~65% | 26/86  (500 nm) | 21.38 (500 nm)  121.03 (1060 nm) | 500 cycles  (2.4% optical loss) |
| ITO/PANI [S5] | | | ~70% | ~80% | 19/6  (500 nm) | 60 (1600 nm) | N/A |
| ITO/PANI  device [S5] | | | ~64.5% | ~56% | N/A | 228 (600 nm)  293 (1600 nm) | N/A |
| W_18_O_49_/TiO_2_ [S6] | | | ~45% | ~65% | NA | NA | 20,000 cycles  (15% capacity loss) |
| Nb-doped TiO_2_ nanocrystals  device [S7] | | | ~76% | ~60% | 105/~10  (500 nm) | N/A | 200 cycles |
| Ta-doped TiO_2_ nanocrystals [S8] | | | ~90% | ~82% | 66.8/6.9  (550 nm) | 33.2 (550 nm)  124.5 (1600 nm) | 2000 cycles  (14.8% capacity loss) |
| TiO_2-x_  Nanocrystals [S9] | | | 95.5% | 90.5% | 35.1/9.6  (633 nm) | 38.2 (633 nm)  112.7 (1600 nm) | 2000 cycles  (4.4% capacity loss) |
| TiO_2-x_  Nanocrystals  device [S9] | | | 74.5% | 73.2% | N/A | N/A | 500 cycles  (10.2% capacity loss) |
| m-WO_3-x_  nanowires [S10] | | | 91.7% | 94.6% | 21/85  （633 nm）  22/75  (1200 nm) | 101.7 (633 nm)  184.3 (1200 nm) | 1000 cycles  (20.0% capacity loss) |
| WO_3_  Nanorods [S11] | | | ~82% | 89% | 98/>1200  （600 nm） | 21 (600 nm)  43 (1200 nm) | 500 cycles  (~16% capacity loss) |
| Nb_12_O_29_ nanoplatelets [S12] | | | 71% | 83% | ~120/~60  （550 nm） | 77.3 (550 nm) | 500 cycles  （32% capacity loss） |
| Nb_18_W_16_O_93_ [S13] | | | 93% | 89% | 10.1/12.7  (633 nm) | 105.6 (633 nm)  113.4 (1200 nm) | 1000 cycles  (10.5% optical loss) |
| MoO_3-x_  nanowires  device [S14] | | | ~75.1% | ~65.8 | 13.7/6.9  (450 nm)  12.4/5.4  (1080 nm) | 211.7 (450 nm)  232.8 (1080) | 1000 cycles  (8.2% CE loss) |
| Li_4_Ti_5_O_12_ [S15] | | | ~64% | ~65% | 55/42  (650 nm)  18/19  (1100 nm) | 8.81 (650 nm)  32.3 (1100 nm) | 500 cycles  (almost unchanged) |
| PANI [S16] | | | 65% | ~61% | 5.9/16.9  (633 nm)  11/32.5  (1600 nm) | 367..1 (633 nm)  299.6 (1600 nm) | 10,000 cycles  (6% optical loss) |
| Ti-doped WO_3_  Nanocrystals [S17] | | | 84.9% | 90.3% | 3.3/25  (633 nm)  6/2.1  (1200 nm) | 114.9 (633 nm)  420.3 (1200 nm) | 1000 cycles  (11% optical loss) |
| WO_3-x_  Nanoflowers [S18] | | | ~34% | ~66% | N/A | N/A | 400 cycles  (2.25% capacity loss) |
| WO_3_/PB [S19] | | | 35.14% | 60.55% | N/A | N/A | 1000 cycles  (almost unchanged) |
| Cs_0.32_WO_3_ [S20] | | | ~65% | 83.7% | 18.4/15.2  (700 nm) | 112.1 (1200 nm) | 10,000 cycles  (no loss) |
| Cs_0.32_WO_3_  device [S20] | | | 65% | 70.8% | 7.6/105.5  (700 nm)  1.5/332.9  (1200 nm) | N/A | N/A |
| VO_2_/WO_3_ [S21] | | | 55%  (670 nm) | 52%  (1500 nm) | 6.2/3.1  (670 nm) | N/A | 1250 cycles  (4.2% capacity loss) |
| **W_18_O_49_**  **nanowires**  **(This work)** | | | **73.1%** | **85.3%** | **14.2/4.8**  **(633 nm) 12/10.8**  **(1200 nm)** | **70.5 (633 nm)**  **185 (1200 nm)** | **N/A** |
| **W_18_O_49_**  **nanowires**  **device**  **(This work)** | | | **51.7%** | **60.0%** | **8.8/15.6**  **(633 nm)**  **5/11.2**  **(1200 nm)** | **52.6 (633 nm)**  **127.9 (1200 nm)** | **10,000 cycles**  **(3.3% capacity loss)** |

**Table S2** Information of the building model applied in actual building energy simulation

| Item | Specifications |
| --- | --- |
| Window fraction (window-to-wall ratio) | 32% of above-grade gross wall |
| Window locations | Even distribution among all four sides |
| Floor to ceiling height | 3.00 m |
| Glazing sill height | 0.7 m |
| Exterior walls | Mass (pre-cast concrete panel): 1IN stucco + 8IN concrete HW + wall insulation + 1/2IN gypsum |
| Roof | Built-up roof: roof membrane + roof insulation + metal decking |
| Window | The window type is described accordingly |
| HVAC | Ideal Loads Air System |
| Personnel | Occupant density:10 m^2^ per person Metabolic rate: 120 W per person |
| Electrical equipment | Equipment heat gain: 15 W m^-2^ |

**Table S3** Optical and infrared properties of the samples used for the simulations

| Parameters | DBED | | | Low-e  glass |
| --- | --- | --- | --- | --- |
|  | Bright mode | Cool mode | Dark mode |  |
| T_sol_ | 0.61 | 0.25 | 0.12 | 0.71 |
| R_sol-Front_ | 0.12 | 0.12 | 0.12 | 0.11 |
| R_sol-Back_ | 0.09 | 0.14 | 0.13 | 0.12 |
| T_VIS_ | 0.64 | 0.45 | 0.24 | 0.83 |
| R_VIS-Front_ | 0.14 | 0.12 | 0.11 | 0.11 |
| R_VIS-Back_ | 0.13 | 0.13 | 0.12 | 0.12 |
| T_IR_ | 0 | 0 | 0 | 0 |
| ε_IR-Front_ | 0.93 | 0.93 | 0.93 | 0.84 |
| ε_IR-Back_ | 0.93 | 0.93 | 0.93 | 0.17 |

**Table S4** Climate zones of various cities around the world [S22], and heating/cooling energy consumption and saving energy consumption vs commercial low-e glass of building for different climate zones.

| Climate zone name | Country | City | Optimal DBED | Low-e glass | Saving energy consumption (MJ m^-2^) | Saving energy percent (%) |
| --- | --- | --- | --- | --- | --- | --- |
| AF | Singapore | Singapore | 446.70 | 563.92 | 117.22 | 20.79 |
| AW | Brazil | Brasilia | 344.19 | 493.64 | 149.46 | 30.28 |
| AM | America | Honolulu | 421.98 | 588.41 | 166.43 | 28.28 |
| AM | America | Miami | 408.50 | 557.81 | 149.31 | 26.77 |
| BWK | China | Turpan | 403.08 | 451.74 | 48.67 | 10.77 |
| BWH | Egypt | Cairo | 368.85 | 522.91 | 154.06 | 29.46 |
| BSK | America | Denver | 314.65 | 447.70 | 133.05 | 29.72 |
| BSK | Spain | Zaragoza | 262.85 | 392.61 | 129.76 | 33.05 |
| BSH | America | Phoenix | 445.94 | 624.25 | 178.31 | 28.56 |
| CFA | China | Nanjing | 288.11 | 351.95 | 63.85 | 18.14 |
| CSB | America | San  Francisco | 220.69 | 379.65 | 158.95 | 41.87 |
| CFB | Iceland | Reykjavik | 276.57 | 272.15 | -4.41 | -1.62 |
| DWB | China | Harbin | 508.90 | 495.34 | -13.56 | -2.74 |
| DWA | China | Beijing | 317.53 | 351.01 | 33.48 | 9.54 |
| DFB | Germany | Berlin | 239.50 | 278.49 | 38.98 | 14.00 |
| DFB | Russia | Moscow | 397.64 | 387.55 | -10.09 | -2.60 |
| DWB | Canada | Calgary | 410.15 | 446.80 | 36.65 | 8.20 |
| DFA | America | Boston | 275.76 | 315.46 | 39.70 | 12.58 |

**Supplementary References**

1. L. lordés, G. Garcia, J. Gazquez, D.J. Milliron, Tunable near-infrared and visible-light transmittance in nanocrystal-in-glass composites. Nature **500**, 323–326 (2013). <https://doi.org/10.1038/nature12398>
2. S. Heo, J. Kim, G.K. Ong, D.J. Milliron, Template-free mesoporous electrochromic films on flexible substrates from tungsten oxide nanorods. Nano Lett. **17**, 5756–5761 (2017). <https://doi.org/10.1021/acs.nanolett.7b02730>
3. Z. Wang, Q. Zhang, S. Cong, Z. Chen, J. Zhao et al., Using intrinsic intracrystalline tunnels for near‐infrared and visible‐light selective electrochromic modulation. Adv. Opt. Mater. **5**, 1700194 (2017). <https://doi.org/10.1002/adom.201700194>
4. H. Gu, C. Guo, S. Zhang, L. Bi, T. Li et al., Highly efficient, near-infrared and visible light modulated electrochromic devices based on polyoxometalates and W_18_O_49_ nanowires. ACS Nano **12**, 559–567 (2018). <https://doi.org/10.1021/acsnano.7b07360>
5. P. Yilmaz, M. Magni, S. Martinez, R. M. Gonzalez Gil, M. D. Pirriera et al., Spectrally selective PANI/ITO nanocomposite electrodes for energy-efficient dual band electrochromic windows. ACS Appl. Energy Mater. **3**(4), 3779-3788 (2020). <https://doi.org/10.1021/acsaem.0c00241>
6. Y. Huang, B. Wang, X. Bai, Y. Han, W. Zhang et al., 3D pine‐needle‐like W_18_O_49_/TiO_2_ heterostructures as dual‐band electrochromic materials with ultrafast response and excellent stability. Adv. Opt. Mater. 10, 2102399 (2022). <https://doi.org10.1002/adom.202102399>
7. M. Barawi, L. De Trizio, R. Giannuzzi, G. Veramonti, L. Manna et al., Dual band electrochromic devices based on Nb-doped TiO_2_ nanocrystalline electrodes. ACS Nano **11**, 3576-3584 (2017). <https://doi.org/10.1021/acsnano.6b06664>
8. S. Cao, S. Zhang, T. Zhang, J.Y. Lee, Fluoride-assisted synthesis of plasmonic colloidal Ta-doped TiO_2_ nanocrystals for near-infrared and visible-light selective electrochromic modulation. Chem. Mater. **30**, 4838-4846 (2018). <https://doi.org/10.1021/acs.chemmater.8b02196>
9. S. Zhang, S. Cao, T. Zhang, J.Y. Lee, Plasmonic oxygen-deficient TiO_2-x_ nanocrystals for dual-band electrochromic smart windows with efficient energy recycling. Adv. Mater. **32**, 2004686 (2020). <https://doi.org/10.1002/adma.202004686>
10. S. Zhang, S. Cao, T. Zhang, Q. Yao, A. Fisher et al., Monoclinic oxygen-deficient tungsten oxide nanowires for dynamic and independent control of near-infrared and visible light transmittance. Mater. Horiz. **5**, 291–297 (2018). <https://doi.org/10.1039/C7MH01128H>
11. R. Giannuzzi, R. Scarfiello, T. Sibillano, C. Nobile, V. Grillo et al., From capacitance-controlled to diffusion-controlled electrochromism in one-dimensional shape-tailored tungsten oxide nanocrystals. Nano Energy **41**, 634-645 (2017). <https://doi.org/10.1016/j.nanoen.2017.09.058>
12. H.C. Lu, S. Ghosh, N. Katyal, V.S. Lakhanpal, I.R. Gearba-Dolocan et al., Synthesis and dual-mode electrochromism of anisotropic monoclinic Nb_12_O_29_ colloidal nanoplatelets. ACS Nano **14**, 10068-10082 (2020). <https://doi.org/10.1021/acsnano.0c03283>
13. G. Cai, R. Zhu, S. Liu, J. Wang, C. Wei et al., Tunable intracrystal cavity in tungsten bronze‐like bimetallic oxides for electrochromic energy storage. Adv. Energy Mater. **12**, 2103106 (2021). <https://doi.org/10.1002/aenm.202103106>
14. T. Rao, Y. Zhou, J. Jiang, P. Yang, X. Wang et al., Fluoride-assisted preparation of plasmonic oxygen-deficient MoO_3−x_ nanowires for dual-band electrochromic smart windows. J. Electrochem. Soc. **169**, 066506 (2022). <https://doi.org/10.1149/1945-7111/ac741e>
15. T. Bai, W. Li, G. Fu, Y. Shen, Q. Zhang et al., Dual-band electrochromic optical modulation improved by a precise control of lithium content in Li_4+x_Ti_5_O_12_. ACS Appl. Mater. Interfaces **14**, 52193-52203 (2022). <https://doi.org/10.1021/acsami.2c16654>
16. Q. Wang, S. Cao, Q. Meng, K. Wang, T. Yang et al., Robust and stable dual-band electrochromic smart window with multicolor tunability. Mater. Horiz. **10**(3), 960-966 (2023). <https://doi.org/10.1039/d2mh01365g>
17. Q. Meng, S. Cao, J. Guo, Q. Wang, K. Wang et al., Sol-gel-based porous Ti-doped tungsten oxide films for high-performance dual-band electrochromic smart windows. J. Energy Chem. **77**, 137-143 (2023). <https://doi.org/10.1016/j.jechem.2022.10.047>
18. Y. Huang, B. Wang, P. Lyu, S. Zhao, X. Wu et al., Oxygen-deficient tungsten oxide nanoflowers for dynamically tunable near-infrared light transmittance of smart windows. Nano Res. **16**, 12165–12172 (2023). <https://doi.org/10.1007/s12274-023-5600-7>
19. S. Zhao, B. Wang, Y. Huang, Y. Zhang, X. Wu et al., WO_3_ nanorod array-modified prussian blue with long cycling stability for high-performance dual-band electrochromic materials. ACS Appl. Nano Mater. **6**, 15021-15028 (2023). <https://doi.org/10.1021/acsanm.3c02567>
20. F. Zhao, C. Li, S. Li, B. Wang, B. Huang et al., Continuous solar energy conversion windows integrating zinc anode‐based electrochromic device and IoT system. Adv. Mater. **36**, 2405035 (2024). <https://doi.org/10.1002/adma.202405035>
21. Z. Shao, A. Huang, C. Cao, X. Ji, W. Hu, H. Luo, J. Bell, P. Jin, R. Yang, X. Cao. Tri-band electrochromic smart window for energy savings in buildings. Nat. Sustain. **7**, 796-803 (2024). <https://doi.org/10.1038/s41893-024-01349-z>
22. H. Zhang, X. Zhang, W. Sun, M. Chen, Y. Xiao et al., All‐solid‐state transparent variable infrared emissivity devices for multi‐mode smart windows. Adv. Funct. Mater. **34**, 2307356 (2023). <https://doi.org/10.1002/adfm.202307356>
